# Transcriptome Analysis of Early Surface-Associated Growth of *Shewanella oneidensis* MR-1

**DOI:** 10.1371/journal.pone.0042160

**Published:** 2012-07-31

**Authors:** Julia Gödeke, Lucas Binnenkade, Kai M. Thormann

**Affiliations:** Department of Ecophysiology, Max-Planck-Institut für terrestrische Mikrobiologie, Marburg, Germany; Université d’Auvergne Clermont 1, France

## Abstract

Bacterial biofilm formation starts with single cells attaching to a surface, however, little is known about the initial attachment steps and the adaptation to the surface-associated life style. Here, we describe a hydrodynamic system that allows easy harvest of cells at very early biofilm stages. Using the metal ion-reducing gammaproteobacterium *Shewanella oneidensis* MR-1 as a model organism, we analyzed the transcriptional changes occurring during surface-associated growth between 15 and 60 minutes after attachment. 230 genes were significantly upregulated and 333 were downregulated by a factor of ≥2. Main functional categories of the corresponding gene products comprise metabolism, uptake and transport, regulation, and hypothetical proteins. Among the genes highly upregulated those implicated in iron uptake are highly overrepresented, strongly indicating that *S. oneidensis* MR-1 has a high demand for iron during surface attachment and initial biofilm stages. Subsequent microscopic analysis of biofilm formation under hydrodynamic conditions revealed that addition of Fe(II) significantly stimulated biofilm formation of *S. oneidensis* MR-1 while planktonic growth was not affected. Our approach to harvest cells for transcriptional analysis of early biofilm stages is expected to be easily adapted to other bacterial species.

## Introduction

The majority of bacteria in nature are thought to exist in biofilms, surface-associated communities in which the cells are embedded within a self-produced matrix [1]. Numerous studies have provided evidence that cells in biofilms exhibit drastically altered properties compared to their planktonic counterparts. Particularly due to their enhanced tolerance towards all kinds of environmentally induced stresses, biofilms are important in environmental, medical, and industrial settings [2], [3]. Biofilm formation is a complex process which significantly varies between bacterial species and depends on the environmental conditions, however, it is generally divided into several commonly occurring steps: Planktonic cells attach to a surface, become permanently immobilized, and begin to form three-dimensional structures [4], [5]. Factors such as flagella-mediated motility and proteinaceous surface structures, pili or fimbriae, are thought to facilitate surface contact and to mediate transient attachment [6]. Permanent attachment might require further adhesins, such as LapA in *Pseudomonas fluorescens* [7], or the polysaccharide PGA in *Escherichia coli* [8]. A recent study demonstrated that production of the polar holdfast adhesive polysaccharide required for permanent attachment of *Caulobacter crescentus* is stimulated by surface contact, a process that occurred within minutes [9]. Thus, the response of bacterial cells to surface attachment is an important prerequisite for subsequent biofilm development. In contrast, bacterial association with a surface might not only lead to a sessile lifestyle but may result in another form of group behavior, a flagella-mediated movement of cell groups over a surface, referred to as swarming [10]. However, the nature and perception of underlying signals and the corresponding regulating systems involved in stimulating either biofilm formation or swarming are still largely unknown.

Dissimilatory metal ion-reducing bacteria are involved in the elemental cycling of metals and are thought to bear potential for applications in bioremediation processes or the production of microbial fuel cells [11]–[13]. The facultatively anaerobic gammaproteobacterium *Shewanella oneidensis* MR-1 has become a model organism for biofilm formation of species belonging to this group of bacteria. A number of studies have been carried out by monitoring surface-associated growth of fluorescently labeled cells in hydrodynamic flow chamber systems by means of confocal laser scanning microscopy. The studies have demonstrated that biofilm formation of *S. oneidensis* MR-1 starts with single cells attaching to the surface and proceeds through formation of microcolonies and surface coverage before large three-dimensional structures are formed [14]–[24]. A functional flagellar system, a mannose-sensitive heme agglutination (MSHA) type IV pilus system, and the presence of extracellular DNA (eDNA) are implicated to be critical factors involved in the initial step [15], [17], [20]. The following progression from the monolayer state to three-dimensional development depends on the *mxdABCD* gene cluster, which is likely required for the synthesis of an extracellular polysaccharide as an important component for the structural integrity of the population [16], [20]. In addition, eDNA and a Bap/RTX cell surface protein have been demonstrated to be required for normal biofilm formation [15], [25].

To characterize the acclimation to surface-associated growth of *S. oneidensis* MR-1, we intended to perform global transcriptomic analysis of surface-associated cells by microarrays. This powerful technique has been used to study biofilm formation in a wide array of bacterial species [26]. A recent study provided a transcriptional analysis of *S. oneidensis* MR-1 growing on the surface of an electrode compared to planktonic cells using ferric citrate or oxygen as terminal electron acceptor [27]. However, to best of our knowledge, global transcriptomic studies on the very early biofilm phases directly following surface attachment have not been published so far for this or any other bacterial species. A major obstacle is the low amount of cell material that is attached during the initial stages and a proper separation of attached and non-attached cells. Thus, a cell harvesting system was required that would exhibit the following features: i) It should be a hydrodynamic system to reliably separate attached from non-attached cells and to mimic flow cell conditions. The latter would then allow subsequent microscopic characterization of potential factors identified by transcriptomic analysis in the flow cell system. ii) It should allow rapid cell harvest under standardized conditions to avoid a long handling time which might result in degradation of unstable RNA species and to yield comparable results between different experiments. iii) It should be simple to set up to facilitate the harvest of sufficient cell material for subsequent studies without the necessity of RNA amplification. Here, we describe an appropriate cell harvesting system that meets all of the above requirements. We applied this system to analyze for the first time the transcriptomic changes occurring during early phases of bacterial surface-associated growth under hydrodynamic conditions with *S. oneidensis* MR-1 as model organism. Subsequent microscopic studies using a flow chamber system confirmed that the approach identified novel factors involved in early attachment of this species.

## Results and Discussion

### Establishing a Hydrodynamic System for the Enrichment of Attached Cells

To create a suitable system to harvest sufficient cells for subsequent transcriptomic analysis, we applied a variation of a “packed-bed reactor” described earlier by Grishin and Tuovinen [28]. To this end, we used 50-ml syringe columns filled with approximately 200 glass beads with 5 mm diameter that served as substratum for cellular attachment (Fig. S1). This fairly large bead diameter was chosen to avoid the formation of voids unaffected by medium flow. 10 ml of medium were required to fully cover the beads. The syringe column was sealed by a rubber stopper and flow of fresh medium from a corresponding medium reservoir was provided by a peristaltic pump through a syringe needle. The medium flow was set to 3.3 ml⋅min^−1^. This high flow rate in concert with the rather small amount of beads/medium was expected to remove non-attached cells and to minimize the formation of gradients with respect to available oxygen, nutrients, and/or secondary metabolites that might be established due to the metabolic activity of the attached cells. To determine turnover of the medium within the system, 10 ml medium stained with crystal violet (0.01%/w/v)) was used as a marker. After 4 minutes of medium flow almost no crystal violet remained in the system as measured spectrophotometrically in the outflow, indicating effective turnover within the system (Fig. S1). Our set-up allowed usage of up to twelve columns in parallel to increase the yield of cell material.

**Figure 1:**
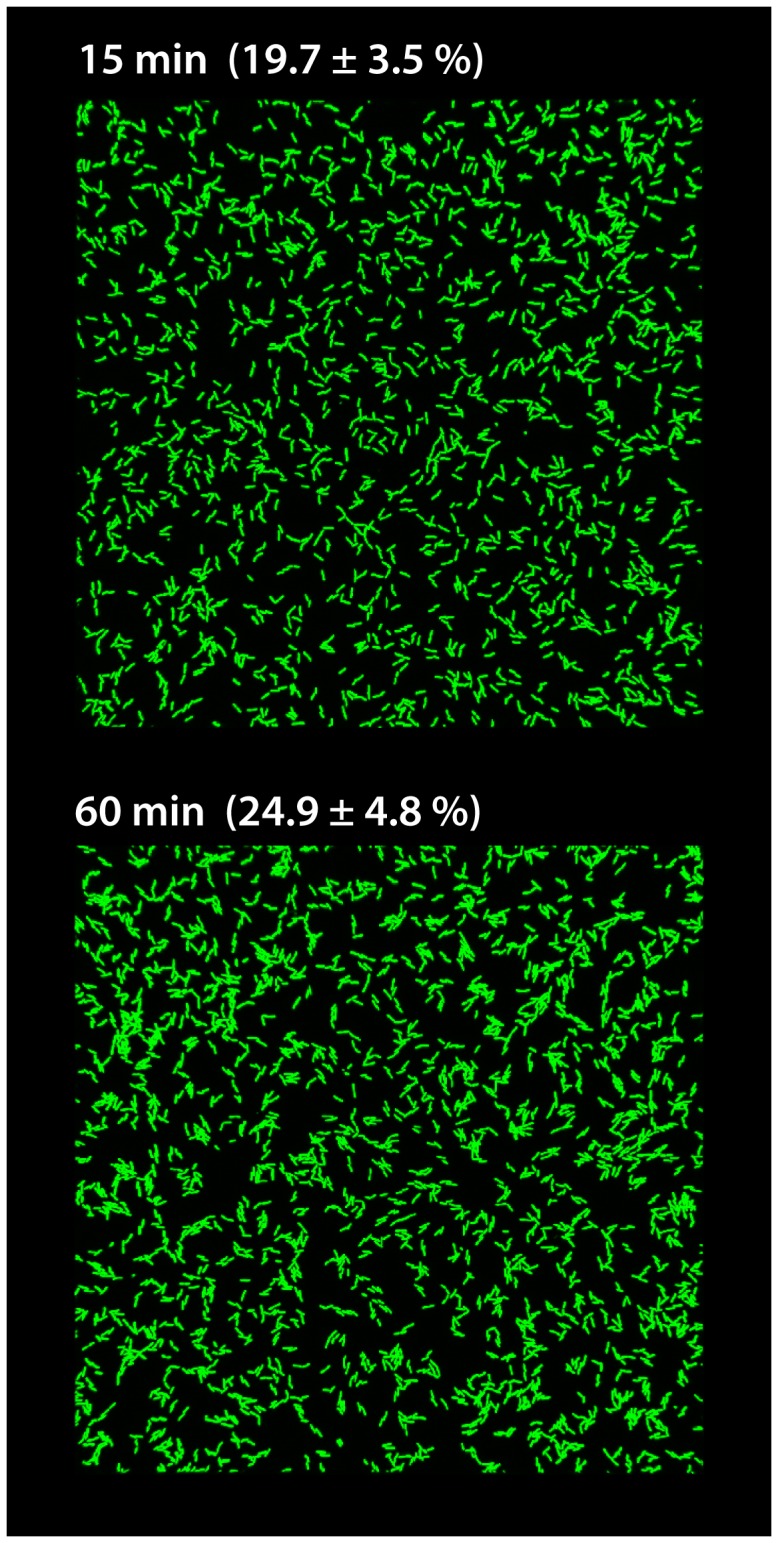
Coverage of a glass surface after inoculation and medium flow for 15 (upper image) and 60 minutes (lower image). Displayed are images taken by confocal laser scanning microscopy in flow chambers at the indicated time points. The lateral edge of each image equals 250 µm. The values given represent the average and standard deviation of surface coverage in percent of 6 randomly selected spots in three different channels of a flow chamber.

A general problem in applying microarray analysis on bacteria during biofilm formation results from the vast heterogeneity of conditions once the cells have started to form three-dimensional structures. Thus, after equilibration, the system was inoculated using 10 ml of a *S. oneidensis* MR-1 cell culture freshly diluted to an OD_600_ of 0.1 and was incubated without flow for 10 minutes. Previous microscopic experiments using flow chambers revealed that under these conditions single cell attachment occurs in a way that about 20% (19.67±3.51%) of the surface is covered (Fig. 1). This rather low cell amount was used to ensure that cell-cell interactions are kept to a minimum and that the formation of steep gradients with respect to oxygen, nutrients, and/or metabolites is prevented. After 10 minutes of attachment, the column was sealed with a rubber stopper and flow was resumed to remove all cells that were not firmly attached. After 60 minutes of incubation the surface coverage only slightly increased (to 24.93±4.83%; Fig. 1). To harvest the cell material the flow was stopped at appropriate time points, the residual medium was discarded through the outflow, and the beads with the attached cells were immediately transferred into stopping solution. The cells were released from the surface by short and thorough vortexing. At least 6.35⋅10^4^ cells could be recovered from each column by this method.

### Transcriptomic Analysis of Early Attachment

Using this cell harvesting system we analyzed the transcriptomic changes in *S. oneidensis* MR-1 following permanent attachment. Our and most other systems do not allow a harvest of planktonic and surface-associated cells under the same conditions. Thus, changes in the transcriptional levels might also be attributed to differences in cultivation or occurring during the process of cell recovery. Therefore, we did not compare the transcriptomes of cells from the planktonic phase to those of attached cells. Instead, for a direct comparison we chose the transcriptomes of cells that have been attached for 15 (as reference) and 60 minutes after resuming the medium flow. At this stage, the cells have firmly attached to the surface, however, formation of three-dimensional structures has not yet started. Thus, our analysis does not aim at the direct transcriptional response to surface attachment but covers the subsequent changes during early surface-associated growth. In order to obtain sufficient material for RNA isolation, the cell material of more than 30 column runs from at least 6 independent experiments was combined. High quality RNA was prepared and used for subsequent microarray analysis.

**Figure 2:**
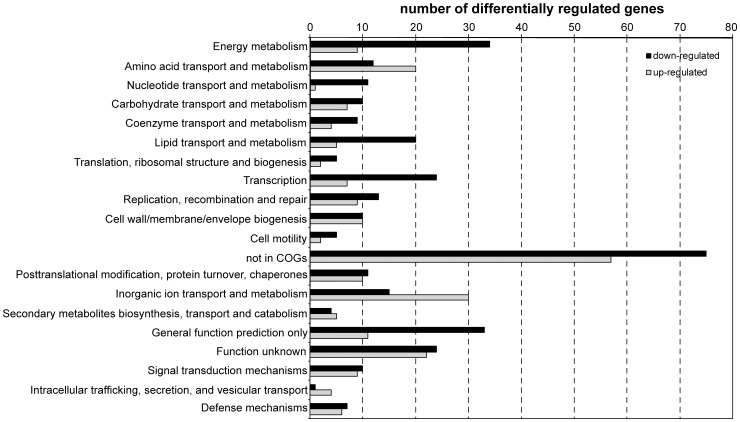
Functional categories of significantly regulated genes comparing cells attached for 60 minutes to cells attached for 15 minutes. Up-regulated genes are displayed in light grey, downregulated genes in black. The only two functional groups that are comprising more genes that are upregulated than downregulated are those of transport and metabolism of amino acids and inorganic ions, in particular iron, respectively.

According to the statistical analysis (*p* value ≤0.05), 563 genes exhibited at least a two-fold change (log_2_ fold change ≥1) in gene expression, representing approximately 11.8% of the 4,770 ORFs spotted on the array. We found 230 genes to be upregulated after 60 minutes compared to 15 minutes after attachment (Table S1), 333 genes were downregulated (Table S2). The reliability of the microarray analysis was evaluated by performing quantitative RT-PCR on selected genes (Fig. S2). The differentially expressed genes of each data set covered all functional categories according to the JCVI annotation (http://cmr.jcvi.org) (Fig. 2), indicating that the adaptation to surface-associated growth causes global changes at the transcriptional level. As has been observed in numerous other microarray studies on bacterial biofilms, genes with unknown function or that do not belong to a certain functional category represent the by far largest group that comprises more than 30% of all significantly regulated genes. Notably, genes of most systems that have been previously demonstrated to be required for initial attachment as well as later stages of biofilm formation are not significantly regulated during that stage: Expression of the flagellar system is almost not affected, the genes encoding the components of both type IV pilus systems maintain levels of transcription, and also the MxdABCD and RTX toxin export systems are neither up- nor downregulated. The MSHA pilus was demonstrated to be a prerequisite for attachment but also for initial three-dimensional growth during biofilm formation of *S. oneidensis* MR-1 [17], [20], and, thus, is likely to be permanently expressed during the initial phases. Similarly, a functional flagellar system has been shown to be an important factor through all stages of *S. oneidensis* MR-1 biofilm formation [17]. The putatively exopolysaccharide-producing MxdABCD system is required for the progression from the monolayer stage to extensive three-dimensional growth [17], [20]. Our study suggests that Mxd is not upregulated at that early stage, in contrast to holdfast EPS production in *Caulobacter crescentus* or alginate formation in *Pseudomonas aeruginosa* [9], [29]. Taken together, these findings imply that factors involved in three-dimensional growth are already present or not yet activated at that early stage of surface attachment. This is consistent with findings reported by Rosenbaum *et al*. [27] who also observed that in monolayer biofilms of *S. oneidensis* MR-1 grown for 14 days on an electrode the *mxd* cluster is also not induced compared to planktonic cells. However, there is generally little overlap in the transcriptomic changes identified in this and our study. This is not surprising since Rosenbaum *et al*. compared the transcriptomes of planktonic and surface-associated cells, and the biofilms have been grown and harvested under significantly different conditions [27].

### eDNA Production and Turnover

A previous study by our group has provided evidence that extracellular DNA (eDNA) is an important structural component for normal biofilm formation of *S. oneidensis* MR-1 [15]. This study strongly indicated that at least two of the three prophages encoded in the genome of this species, LambdaSO and MuSO2, are playing major roles in cell lysis that might be required to release DNA and other factors that contribute to cell-cell and cell-surface interactions. Notably, genes of both LambdaSO and MuSO2 are significantly regulated during early surface growth (Table S3). While Lambda appears to be induced, MuSO2 was downregulated which was rather unexpected since previous experiments indicated that MuSO2 is induced during early stages of biofilm formation. A putative holin/antiholin system (SO_1046– SO_1048), that has previously been identified in *S. oneidensis* MR-1 [30], is also downregulated, indicating that holin-mediated lysis of *S. oneidensis* MR-1 cells is suppressed at that stage. Interestingly, SO_1066, encoding the extracellular nuclease ExeM, is already upregulated during early surface-associated growth. This nuclease has been identified to be required for turnover of eDNA in the biofilm matrix, and the absence of ExeM results in eDNA accumulation and tight cell-cell interactions [14], [31]. Thus, upregulation of *exeM* at that early stage of surface growth emphasizes the observed role of the nuclease in *Shewanella* biofilm formation and indicates a function for nucleolytic activity already at the surface-adhesion state.

### Metabolic Adaptation

A number of transcriptional changes affect genes encoding components of metabolism. Among those are genes mediating the transport and conversion of carbohydrates (Table S4) that are upregulated (SO_2486– SO_2491, *eda-edd-pgl-zwf-pykA*; SO_3547, *pgi*). This group includes the key components of the Entner-Doudoroff pathway involved in gluconate catabolism, 6-phosphogluconate dehydratase (Edd) and 2-keto-3-deoxy-6-phosphogluconate (KDPG) aldolase (Eda) [32], [33]. Lipid metabolism is downregulated (Table S5) along with a number of genes involved in butanoate metabolism as well as genes involved in purine metabolism (Table S6) and, associated, thiamine synthesis. A large fraction of significantly regulated genes belongs to the functional category of amino acid transport and metabolism (Table 1) and include an upregulation of tryptophan synthesis (SO_3024– SO_3019, *trpABCFGE*; SO_1367, *pheA*) and part of the valine synthesis (SO_4344, *ilvA*; SO_4346, *ilvM*; SO_4349, *ilvC*). The regulation of these defined sets of genes indicates a specific rather than a global response with respect to metabolism during the early surface-associated phase. The upregulation of tryptophan synthesis was particularly interesting since this pathway is also involved in production of indole, which has been demonstrated to act as intercellular signaling molecule in a number of bacterial species [34]. Indole is implicated in regulating a number of cellular processes and has been shown to affect biofilm forming bacterial species such as *Escherichia coli* [35]–[38], *P. aeruginosa* [38], [39], or *Vibrio cholerae* [40]. Notably, a transcriptomic analysis of *E. coli* biofilm formation revealed an upregulation of tryptophan biosynthesis after 4 and 7 hours of incubation, before it was downregulated [41]. However, *S. oneidensis* (as well as most other *Shewanella* species) lack a clear homolog to the tryptophan-indol lyase TnpA, the enzyme that converts tryptophan to indol, and, accordingly, *Shewanella* species that were tested so far do not produce significant amounts of this compound. Addition of tryptophan to the culture medium did not result in any visible changes in biofilm formation at early or later stages (data not shown). However, it cannot be excluded that *S. oneidensis* produces a tryptophan-based signaling molecule that might be relevant in earlier stages of biofilm formation. This might be particularly relevant since species of *Shewanella* are unlikely to produce homoserine lactones, and autoinducer 2, although exported in earlier stages of growth, appears to play a minor if any role in quorum sensing in *S. oneidensis* MR-1 [24].

**Table 1:** Differentially regulated genes related to “metabolic adaptation” – amino acid transport and metabolism.

Locus	Gene	Product	log_2_ ratio
trytophan			
SO 3019	*trpE*	anthranilate synthase component I	1.02
SO 3020	*trpG*	anthranilate synthase component II	1.45
SO 3022	*trpC/F*	bifunctional indole-3-glycerol phosphate synthase/phosphoribosylanthranilate isomerase	1.13
SO 3023	*trpB*	tryptophan synthase subunit beta	1.46
SO 3024	*trpA*	tryptophan synthase subunit alpha	1.77
SO 1367	*pheA*	chorismate mutase/prephenate dehydratase	1.17
threonine			
SO 3413	*thrC*	threonine synthase	1.65
SO 4344	*ilvA*	threonine dehydratase	1.22
SO 4349	*ilvC*	ketol-acid reductoisomerase	1.63
others			
upregulated			
SO 0858	*–*	sodium:alanine symporter family protein	2.46
SO 0919	*–*	serine transporter, putative	1.13
SO 2248	*sdaA*	L-serine dehydratase 1	1.05
SO 3142	*dcp-1*	peptidyl-dipeptidase Dcp	1.36
SO 4618	*–*	prolyl oligopeptidase family protein	1.46
SO A0048	*–*	prolyl oligopeptidase family protein	1.32
downregulated			
SO 0275	*argC*	N-acetyl-gamma-glutamyl-phosphate reductase	−1.34
SO 0781	*gcvP*	glycine dehydrogenase	−1.25
SO 1812	*mdeA*	methionine gamma-lyase	−1.27
SO 1893	*mvaB*	hydroxymethylglutaryl-CoA lyase	−1.32
SO 3727	*cysD*	sulfate adenylyltransferase subunit 2	−2.29
SO 3986	*lysC*	aspartate kinase III	−1.65

### An Effect of Iron on S. Oneidensis MR-1 Biofilm Formation

Another large group of genes that were found to be significantly downregulated belong to the functional group involved in energy metabolism (Table S7). Many of these genes encode proteins that are implicated in anaerobic growth, including the nitrate reductase system (SO_0849–SO_0845, *napDAGHB*) and the formate dehydrogenase complexes (SO_0101–SO_0107, *fdnGHIE selAB fdhD*; SO_4513–SO_4515, *fdhA_2_B_2_C_2_*), and a Ni/Fe hydrogenase (SO_2096–2098, *hydBC*). This finding indicates that the hydrodynamic system is a highly aerobic environment that does not require anaerobic metabolic activity. However, it was unexpected that such enzymatic systems are further downregulated in later stages of surface attachment since the increase in the number of cells would assumed to rather cause a lack of oxygen. A second possibility is that inactivation of the anaerobic respiration systems releases iron that otherwise would be sequestered in many of the proteins involved [42], [43]. This in agreement with the observation that many of the genes belonging into the functional category of inorganic ion transport and metabolism rank among the most highly regulated genes in this study, and among those are numerous genes encoding for known or potential systems involved in iron acquisition and uptake (Table 2). A majority of these systems have previously been identified in a recent study in which changes in expression profiles of *S. oneidensis* MR-1 under iron depletion were determined [44]. These include a large gene cluster encoding a TonB system and hemin ABC transporter (SO_3665 - SO_3675; TonB1-ExbB1-ExbD1 and *hmuTUV*) as well as a gene cluster encoding the synthesis of the siderophore alcaligin (SO_3030– SO_3033; AlcABC). Other genes that are thought to be involved in the *S. oneidensis* iron response are SO_0139 (*ftn*, ecoding ferritin), SO_1482, SO_3914, and SO_4743 (encoding putative TonB-dependent receptor proteins), or the ferric vibriobactin and enterobactin receptors (SO_4516, *viuA*; SO_4523, *irgA*). With respect to the synchronous downregulation of iron-sequestering gene products such as electron transport components MtrB (SO_1776) and OmcB (SO_1778), or the ferrochelatase HemH-1 (SO_2019), this finding strongly indicates a high demand of the cells for iron during the adaptation phase to surface-associated growth. Elevated acquisition of iron might result in oxidative stress [45], and, accordingly, corresponding defense systems are induced (SO_0725, *catG1*; SO_1070, *katB*; SO_0956/SO_0958, *ahpF*/*ahpC*, encoding subunits of a peroxidase, a catalase, and an alkyl hydroperoxide reductase). In addition, the downregulation of sulfate transport, which would be required to rebuild Fe-S centers, as well the absence of an induction of DNA repair systems indicate that the iron acquisition response to surface attachment is not caused by cellular repair processes to recover from extensive oxidative stress.

**Table 2:** Differentially regulated genes potentially related to iron homeostasis.

Locus	Gene	Product		log_2_ ratio
Iron transport and storage				
SO 0139	*ftn*	ferritin		1.80
SO 0630	*nosA*	TonB-dependent receptor		1.51
SO 1482	*–*	TonB-dependent receptor, putative		5.02
SO 1580	*–*	TonB-dependent heme receptor		2.42
SO 2907	*–*	TonB-dependent receptor domain-containing protein		1.56
SO 3030	*alcA*	siderophore biosynthesis protein		5.33
SO 3031	*–*	siderophore biosynthesis protein (AlcB)		3.94
SO 3032	*–*	siderophore biosynthesis protein (AlcC)		5.81
SO 3033	*–*	ferric alcaligin siderophore receptor (AlcD)		5.61
SO 3667	*–*	heme iron utilization protein		3.62
SO 3668	*–*	HugX family protein		3.64
SO 3669	*hugA*	heme transport protein		2.71
SO 3670	*tonB1*	TonB1 protein		3.51
SO 3671	*exbB1*	TonB system transport protein ExbB1		5.70
SO 3672	*exbD1*	TonB system transport protein ExbD1		5.38
SO 3673	*hmuT*	hemin ABC transporter, periplasmic hemin-binding protein		4.04
SO 3674	*hmuU*	hemin ABC transporter, permease protein		2.33
SO 3675	*hmuV*	hemin importer ATP-binding subunit		5.03
SO 3914	*–*	TonB-dependent receptor, putative		3.15
SO 4077	*–*	TonB-dependent receptor, putative		4.70
SO 4516	*viuA*	ferric vibriobactin receptor		3.60
SO 4523	*irgA*	enterobactin receptor protein		1.80
SO 4743	*–*	TonB-dependent receptor, putative		1.76
downregulated				
SO 2019	*hemH-1*	ferrochelatase	−1.58	
Oxidative stress				
SO 0725	*katG-1*	catalase/peroxidase HPI		1.47
SO 1070	*katB*	catalase		1.37
SO 0956	*ahpF*	alkyl hydroperoxide reductase, F subunit		2.38
SO 0958	*ahpC*	alkyl hydroperoxide reductase, C subunit		2.22

**Figure 3:**
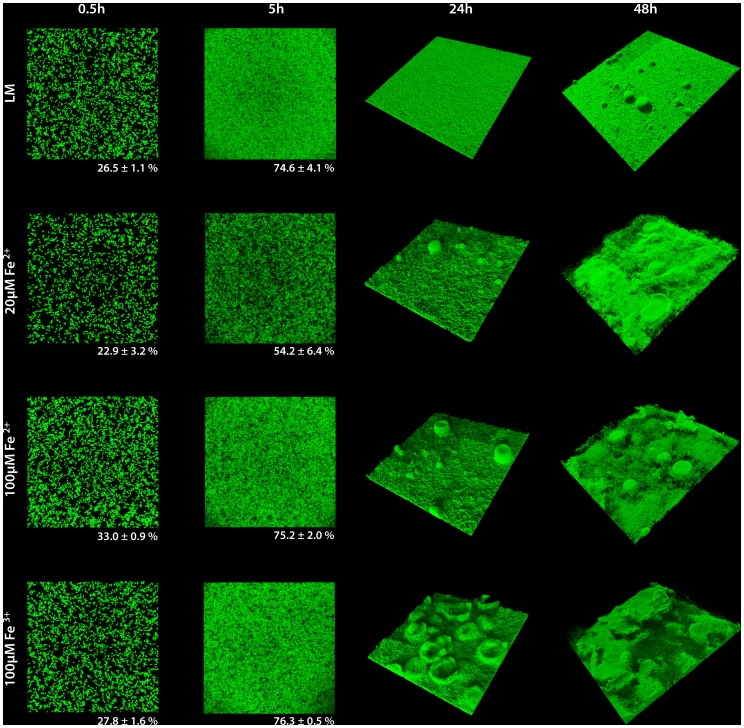
Influence of iron addition to *S. oneidensis* MR-1 biofilm formation under hydrodynamic conditions. Gfp-tagged *S. oneidensis* MR-1 wild-type cells were incubated in flow chambers in LM medium (upper panel) that was supplemented with iron at the indicated concentrations (lower panels). Biofilm formation was analyzed by CLSM at the indicated time points, displayed are three-dimensional shadow projections. The numbers below the images recorded after 0.5 and 5 hours represent the average surface coverage. The lateral edge of each image is 250 µm in length.

**Figure 4:**
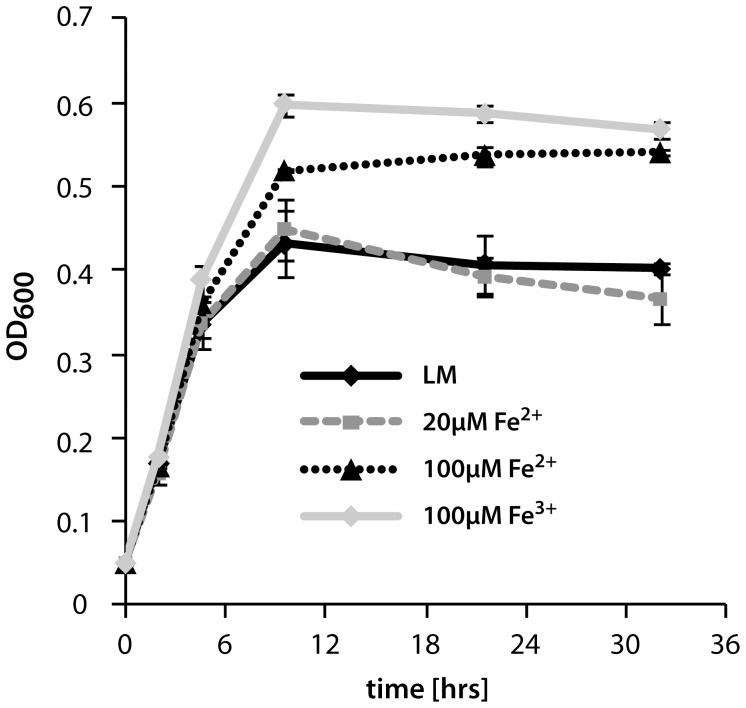
Influence of iron addition to planktonic growth of *S. oneidensis* MR-1 in LM medium. Addition of 100 µm Fe^2+^ or Fe^3+^ leads to higher end oD. The values represent the average of 6 independent growth experiments, the error bars represent the standard deviation.

To further determine a potential role of iron in *S. oneidensis* MR-1 biofilm formation, we characterized the effect of different iron levels in the hydrodynamic flow chamber system (Fig. 3). While addition of 20 µM FeCl_2_ to the medium did not result in any differences in planktonic growth (Fig. 4), a significant biofilm phenotype occurred in flow cells. The coverage of the surface was delayed in earlier stages (22.9% to 26.5% after 30 minutes and 54.2% to 74.6% after 5 hours), and the formation of three-dimensional structures occurred earlier and more structures were formed. This effect was observed in a more pronounced fashion upon addition of 100 µM FeCl_2_ or FeCl_3_. However, at these concentrations the cells also reached higher optical densities in planktonic cultures. Beneficial effects on planktonic growth or biofilm formation were not observed when equimolar amounts of MnCl_2_ were added (data not shown). The results indicate that the high demand of iron during early stages of surface-associated growth as identified by our transcriptomic analysis is reflected in a biofilm phenotype under similar conditions.

Iron is an essential co-factor of proteins that are required for many basic and metabolic functions in bacteria. There is growing evidence that the available level of this element drastically affects bacterial group behaviors as has been demonstrated for a number of species such as *Acinetobacter baumannii*, *Legionella pneumophila*, *Staphylococcus aureus*, or *Streptococcus mutans*, or *V. cholerae* [46]–[50]. The best studied organism with respect to the role of iron in biofilm formation is *P. aeruginosa*. In this species, iron limitation or impairment in iron uptake strongly inhibits the formation of three-dimensional structures but rather favors pili-mediated twitching motility across the surface [51]–[54]. Another study suggests that an addition of low amounts of iron stimulates biofilm formation through expression of the *pqs* genes, required for the formation of the 2-alkyl-4-quinolone signal molecule PQS, and DNA release [55], [56]. It has been hypothesized that this behavior might help the cells of *P. aeruginosa* to move to locations that offer sufficient supply of iron to meet the requirements for biofilm formation [51]. Notably, surface motility by flagella-mediated swarming is similarly stimulated by iron limitation in *E. coli* and *Vibrio parahaemolyticus* [57]–[59], and during swarming of *P. aeruginosa* iron acquisition is downregulated in the fast moving cells at the tendril tips [60]. Thus, the availability of iron appears to be a common signal for microbial group behaviors at surfaces, as in most environments microbes have to compete for this important nutrient due to the low solubility of Fe(III). For pathogens this strategy might ensure that enough iron is stored to allow growth in or in close vicinity of their corresponding host. In *S. oneidensis* MR-1, an ample amount of iron might be required to establish the electron transport systems that allow cells deep within biofilms to access potential terminal electron acceptors at the surface of the community or at a redox-active substratum [61]. Accordingly, a recent study has demonstrated that iron acquisition systems are also upregulated in pellicles formed by *S. oneidensis* MR-1 at air-liquid interfaces [62].

### Regulatory Components

A fairly large number of differentially regulated genes encode proteins that are implicated to play a role in signal transduction and/or transcription (Table 3), most of which are uncharacterized so far. Not surprisingly, some of these (SO_1415, SO_2005, SO_2039, and SO_3297) have been previously identified to be among the genes regulated in the response to iron starvation and repletion. In the same study SO_1415, a transcriptional regulator of the TetR family, was demonstrated to be involved in anaerobic iron reduction [44]. SO_2426, endcoding a CpxR-like two-component response regulator, is part of the ferric uptake regulator (Fur) regulon in *S. oneidensis* MR-1 [42] and controls expression of genes involved in transport and iron homeostasis [63]. A second homologue to a CpxR-like protein is among the induced regulator components, which likely represents the response regulator of the CpxRA two-component system in *Shewanella*. In *E. coli*, this system is implicated in sensing membrane-associated stress [64] and has been directly linked to biofilm formation of this species through expression control of CsgD, a protein involved in regulating type I fimbriae and cellulose production [65]–[68]. Notably, the protein exhibiting the highest homology to CsgD in *Shewanella*, SO_0864, is also induced after prolonged surface attachment. So far, a role of both regulators in *Shewanella* biofilm formation or other cellular processes has not been demonstrated, a deletion of SO_0864 had no significant effects on biofilm formation (Thormann, unpublished data). However, the results indicate that similar regulatory pathways are active during adaptation to surface growth in both *S. oneidensis* MR-1 and *E. coli* and might respond to similar signals.

**Table 3:** Differentially regulated genes related to regulation.

Locus		Gene	Product		log_2_ ratio
**c-di-GMP production and turnover**					
Upregulated					
SO 0341	–		sensory box protein, GGDEF-EAL		1.33
SO 1208	–		GGDEF-EAL domain-containing protein		1.47
SO 2039	–		hypothetical protein, EAL domain		2.13
SO 2216	–		sensory box protein, GGDEF-EAL		1.35
downregulated					
SO 1695		–	sensory box/GGDEF family protein		−4.39
SO 2498		–	sensory box protein, GGDEF-EAL		−2.11
SO 2174		–	putative cyclic nucleotide phosphodiesterase		−1.58
SO 2366		–	response regulator, HD-GYP domain		−1.48
**LysR family**					
upregulated					
SO 0523		–	LysR family transcriptional regulator		1.27
SO 4524		–	LysR family transcriptional regulator		1.17
downregulated					
SO 0701		–	LysR family transcriptional regulator		−1.04
SO 0843		–	LysR family transcriptional regulator		−1.29
SO 3297		–	LysR family transcriptional regulator		−1.03
SO 3318		–	LysR family transcriptional regulator		−1.00
SO 3874		–	LysR family transcriptional regulator		−1.28
**others**					
upregulated					
SO 0433		*rsd*	anti-RNA polymerase sigma 70 factor		1.11
SO 0864		*–*	LuxR family transcriptional regulator		1.08
SO 1726		*phoU*	transcriptional regulator PhoU		
SO 2426		*–*	DNA-binding response regulator		1.25
SO 4445		*–*	sensor histidine kinase		1.13
SO 4472		*ntrC*	nitrogen regulation protein NR(I)		1.50
SO 4477		*cpxR*	transcriptional regulatory protein CpxR		1.31
downregulated					
SO 0346		–	GntR family transcriptional regulator	−1.36	
SO 0532		*arsR*	arsenic resistance operon repressor	−1.44	
SO 0544		*–*	sensory box histidine kinase	−1.15	
SO 0859		*–*	sensory box histidine kinase/response regulator	−1.64	
SO 0940		*cadC*	transcriptional regulator-related protein	−1.35	
SO 1311		–	transcriptional regulator	−1.33	
SO 1415		–	TetR family transcriptional regulator	−1.41	
SO 1898		–	putative transcriptional regulator	−1.34	
SO 2005		–	dksA-type zinc finger protein	−1.06	
SO 2493		–	TetR family transcriptional regulator	−2.02	
SO 2519		–	AraC family transcriptional regulator	−1.36	
SO 2547		–	response regulator	−1.14	
SO 2653		–	Ner family transcriptional regulator	−2.60	
SO 3385		–	MerR family transcriptional regulator	−3.20	
SO 4624		–	LuxR family transcriptional regulator	−1.19	

Among the significantly regulated genes of this group, eight encode proteins with GGDEF, EAL, and/or HD-GYP domains. These domains are implicated to play a role in synthesis and turnover of c-di-GMP, a secondary messenger molecule generally involved in mediating the transition from planktonic to surface-associated life style in many bacteria [69] and also in *S. oneidensis* MR-1 [16]. This species harbors numerous proteins potentially contributing to c-di-GMP metabolism, and so far only MxdA, a protein with a degenerated GGDEF domain, has been demonstrated to indirectly affect the levels of c-di-GMP and biofilm formation [19]. Interestingly, in contrast to what might be expected, a recent study on *V. parahaemolyticus* strongly indicates that surface adhesion lowers the level of c-di-GMP and favors swarming motility [70]. Thus, further studies need to determine whether or not the genes identified here are involved in biofilm formation of *S. oneidensis* and whether early surface attachment results in an increase or a decrease in intracellular c-di-GMP levels.

### Conclusions

Although numerous microarray studies have aimed at the identification of differences between planktonic and sessile life style, there are no studies that have directly addressed the very early stages following cellular surface attachment. Here, we have applied a simple to use hydrodynamic incubation system that allows the harvest of sufficient amounts of cell material to perform global transcriptomic (or other) analyses without the requirement of subsequent RNA amplification. We have successfully used this system to perform the first transcriptomic analysis of a bacterial response to surface-associated growth. The data will provide starting points for studies on the role of iron acquisition in early biofilm formation. Further studies will also address whether the transcriptomic response of *S. oneidensis* MR-1 is surface-specific.

## Materials and Methods

### Strains, Growth Conditions, and Media

*Shewanella oneidensis* MR-1 wild type [71] and its Gfp-tagged derivative [15] were routinely grown in LB medium at 30°C. When required, media were solidified by adding 1.5% (w/v) agar. Media were supplemented with 6 µg⋅ml^−1^ chloramphenicol, where necessary.

Attachment and biofilm studies of *S. oneidensis* were conducted in LM medium [72] without antibiotics containing 0.5 mM lactate. FeCl_2_ and FeCl_3_ (Merck, Darmstadt, Germany) were used at a concentration of 20–100 µM in LM medium.

### Establishing a Cell Harvesting System

To isolate surface-associated *S. oneidensis* MR-1 cells under hydrodynamic conditions, a suitable harvesting system was established. The system consists of hanging 50 ml-syringe columns (Braun Melsungen AG, Melsungen, Germany) filled with approximately 200 glass beads (5 mm diameter; Roth, Karlsruhe, Germany), which were covered with 10 ml LM medium. The syringe columns were connected via silicone tubes (1⋅3 mm; VWR International GmbH, Darmstadt, Germany) to a medium reservoir and a peristaltic pump (Watson-Marlow 200; Watson-Marlow, Rommerskirchen, Germany) generating hydrodynamic conditions in the system. The setup is displayed in Figure S1. The columns containing the glass beads sealed with rubber stoppers and the silicone tubing were autoclaved separately and assembled afterwards. The medium inflow was connected to the column by a syringe needle passing through the rubber stopper.

To determine the medium turnover within the system, crystal violet was added to the medium reservoir at a final concentration of 0.01% (w/v). Medium flow with fresh medium was started and samples were taken at the outflow in regular time intervals. Presence of crystal violet was measured spectophotometrically at 600 nm.

After calibrating the bead surface with LM medium, overnight cultures of *S. oneidensis* MR-1 grown in LM medium were diluted to an OD_600nm_ of 0.1 and incubated on the glass beads for 10 min prior to starting the medium flow (medium turnover rate 3 ml⋅min^−1^). At desired time points, the cells were harvested by discarding the medium and collecting the glass beads in 15 ml-tubes filled with 1∶10 stopping solution (5% (v/v) phenol (pH 7.4); 95% (v/v) ethanol). The cells were then isolated from the beads by vortexing and centrifuged prior to processing. The glass beads were not reused.

### RNA Isolation

*S. oneidensis* MR-1 cells were harvested by centrifugation at 4,600⋅g for 15 min at 4°C, and the cell sediments were immediately frozen in liquid nitrogen and stored at −80°C. The RNA was extracted from the cells by using the hot phenol method [73]. Residual chromosomal DNA was removed by using Turbo DNA-Free (Applied Biosystems) according to the manufacturer’s instructions. Afterwards, the purified RNA was used for transcriptomic profiling by microarray analysis and quantitative Real Time PCR (q-RT-PCR).

### Microarray Analysis

Microarray analysis was performed with Febit (Febit Biomed GmbH, Heidelberg, Germany). Oligonucleotide probes were synthesized by using light-activated in situ oligonucleotide synthesis inside a biochip of a Geniom One instrument (Febit Biomed GmbH, Heidelberg, Germany) as described previously [74]. Each biochip consists of eight individual microfluidic channels, each of which contained more than 15,000 individual DNA probe features. Using proprietary software from Febit Biomed based on a modified Smith-Waterman algorithm [75], the probe set was calculated from the full *S. oneidensis* MR-1 genome sequence (according to TIGR; accessed on 4^th^ of June, 2008). For each of the 4,770 genes annotated for S. oneidensis MR-1, three 50-mer probes were synthesized. However, for some of the genes, fewer than three probes could be calculated based on the Febit specificity criteria. For each tested condition, two independent pooled RNA samples from at least 6 independent experiments were analyzed. The cDNA labelling, hybridization, array design, and slide scanning were conducted as previously described [76].

### Data Analysis

The resulting detection pictures were evaluated using the Geniom Wizard Software (Febit Biomed GmbH, Heidelberg, Germany). Following background correction, quantile normalization was applied and all further analyses were carried out using the normalized and background subtracted intensity values. The raw data and normalized data are available under Gene Expression Omnibus under accession GSE25865. After having verified the normal distribution of the measured data, parametric t-test (unpaired, two-tailed) was carried out for each gene separately, to detect genes that show a differential expression between the compared groups. The resulting *p* values were adjusted for multiple testing by Benjamini-Hochberg adjustment [77], [78]. For significant statistical measurements, an adjusted *p* value 0.05 (5%) cut-off was applied. All data obtained is MIAME compliant.

### q-RT-PCR

Extracted total RNA was applied as a template for random-primed first-strand cDNA synthesis by using Bioscript (Bioline GmbH, Luckenwalde, Germany) according to the manufacturer’s instructions. The cDNA was used as a template for quantitative PCR (Real-Time 7300 PCR machine; Applied Biosystems Deutschland GmbH, Darmstadt, Germany) by using the Sybr green detection system (Applied Biosystems Deutschland GmbH, Darmstadt, Germany). The cycle threshold (CT) was determined automatically by use of Real-Time 7300 PCR software after 40 cycles. Samples were assayed at least in duplicate. To standardize the quantity of the selected target genes, *recA* served as internal control and was quantified on the same plate as the target genes. Primers used to determine the expression of the corresponding genes are summarized in Table S8. The efficiency of each primer pair was determined by using four different concentrations of *S. oneidensis* MR-1 chromosomal DNA (10 ng⋅l^−1^, 1.0 ng⋅l^−1^, 0.1 ng⋅l^−1^, and 0.01 ng⋅l^−1^) as a template in q-RT-PCRs.

### Cultivation and Imaging of Biofilms

Biofilms were cultivated at room temperature in LM medium in three-channel flow cells with individual channel dimensions of 1 by 4 by 40 mm. Microscope cover slips (Roth, Germany) were used as a colonization surface, glued onto the channels with silicone (Sista-Henkel, Germany), and left to dry for 24 hours at room temperature prior to use. Assembly, sterilization, and inoculation of the flow system were performed essentially as reported earlier [17]. Analyses were carried out in triplicate in at least two independent experiments. When indicated, FeCl_2_ was added to the medium at the desired concentrations. Microscopic visualization of the biofilms and image acquisition were conducted using an inverted Leica TCS SP5 confocal laser scanning microscope (Leica Microsystems, Wetzlar, Germany) equipped with ×10/0.3 Plan-Neofluar and ×63/1.2 W C-Apochromate objectives. CLSM images were processed using the IMARIS software package (Bitplane AG, Zürich, Switzerland).

## Supporting Information

Figure S1 **Set-up and function of the cell harvesting system.** A) The columns consist of bead-filled syringe bodies. The glass beads have a diameter of 5 mm and are covered by 10 ml of medium. B) Medium turnover in the system. Crystal violet (0.01% final concentration) was added to the medium and the oD_600_ of the outflow was measured in regular intervals. At a medium inflow rate of 3.3 ml min^−1^ no crystal violet remained after 4–5 minutes. C) Image of the set-up with the peristaltic pump to the left and a metal rack with the hanging syringe bodies in the centre. Up to 12 columns were run in parallel.
(PDF)

Figure S2 **Validation of the transcriptome data by q-RT-PCR analyses.** The diagrams represent the log_2_-fold changes of the gene expression of SO_2500, SO_2660 and SO_3376 in surface-associated cells under hydrodynamic conditions (harvested after 0.25, 1, 2 and 4 hours) compared to planktonic culture. Plotted on the x-axis are the data values of the microarray analyses, on the y-axis the data values of the q-RT-PCR are shown. The coefficient of determination *R* ^2^ represents the statistical variance of the transcriptome data.
(PDF)

Table S1 Genes significantly upregulated after 60 minutes of attachment compared to 15 minutes of attachment.
(PDF)

Table S2 Genes significantly downregulated after 60 minutes of attachment compared to 15 minutes of attachment.
(PDF)

Table S3 Differentially regulated genes potentially related to cell lysis and eDNA turnover.
(PDF)

Table S4 Differentially regulated genes related to “metabolic adaptation” - Carbohydrate transport and metabolism.
(PDF)

Table S5 Differentially regulated genes related to “metabolic adaptation” - Lipid transport and metabolism.
(PDF)

Table S6 Differentially regulated genes related to “metabolic adaptation” - Nucleotide transport and metabolism.
(PDF)

Table S7 Differentially regulated genes related to “metabolic adaptation” - Energy metabolism and related.
(PDF)

Table S8 Sequence of primers used in this study.
(DOCX)
