# Supplementary material for: Transcriptome Analysis of Early Surface-Associated Growth of Shewanella oneidensis MR-1
Source: PLoS One. 2012 Jul 31;7(7):e42160. doi: 10.1371/journal.pone.0042160 (PMC3409153; doi:10.1371/journal.pone.0042160)
Supplement: Figure S1 — Set-up and function of the cell harvesting system. A) The columns consist of bead-filled syringe bodies. The glass beads have a diameter of 5 mm and are covered by 10 ml of medium. B) Medium turnover in the system. Crystal violet (0.01% final concentration) was added to the medium and the oD600 of the outflow was measured in regular intervals. At a medium inflow rate of 3.3 ml min−1 no crystal violet remained after 4–5 minutes. C) Image of the set-up with the peristaltic pump to the left and a metal rack with the hanging syringe bodies in the centre. Up to 12 columns were run in parallel. (PDF) [file pone.0042160.s001.pdf]

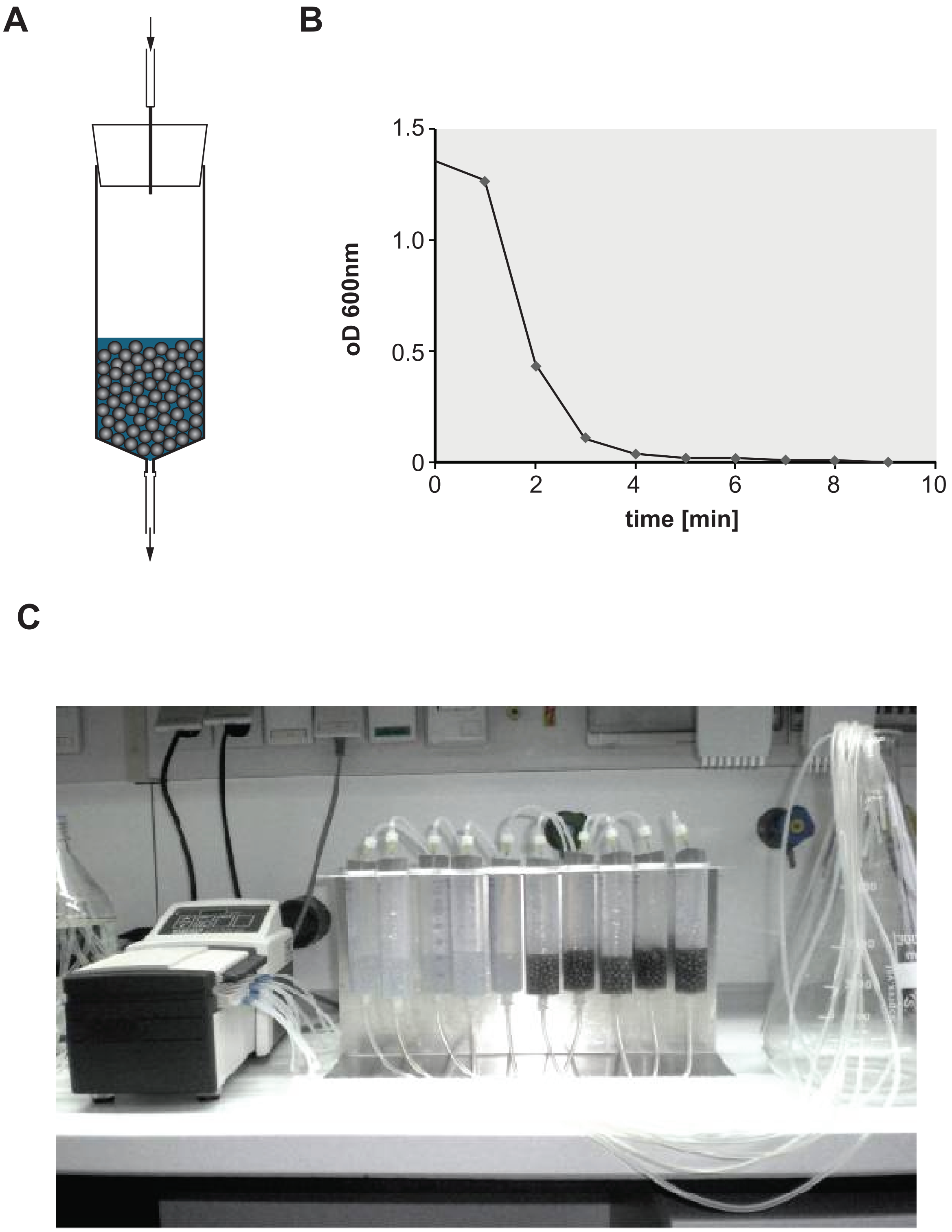

Figure S1: Illustration of the set-up and function of the cell harvesting system. A) The columns consist of bead-filled syringe bodies. The glass beads have a diameter of 5 mm and are covered by 10 ml of medium. B) Medium turnover in the system. Crystal violet (0.01 % final concentration) was added to the medium and the oD600 of the outflow was measured in regular intervals. At a medium inflow rate of 3.3 ml · min-1 no crystal violet remained after 4-5 minutes. C) Image of the set-up with the peristaltic pump to the left and a metal rack with the hanging syringe bodies in the centre. Up to 12 columns were run in parallel.
