# Supplementary material for: Transcriptome Analysis of Early Surface-Associated Growth of Shewanella oneidensis MR-1
Source: PLoS One. 2012 Jul 31;7(7):e42160. doi: 10.1371/journal.pone.0042160 (PMC3409153; doi:10.1371/journal.pone.0042160)
Supplement: Figure S2 — Validation of the transcriptome data by q-RT-PCR analyses. The diagrams represent the log2-fold changes of the gene expression of SO_2500, SO_2660 and SO_3376 in surface-associated cells under hydrodynamic conditions (harvested after 0.25, 1, 2 and 4 hours) compared to planktonic culture. Plotted on the x-axis are the data values of the microarray analyses, on the y-axis the data values of the q-RT-PCR are shown. The coefficient of determination R 2 represents the statistical variance of the transcriptome data. (PDF) [file pone.0042160.s002.pdf]

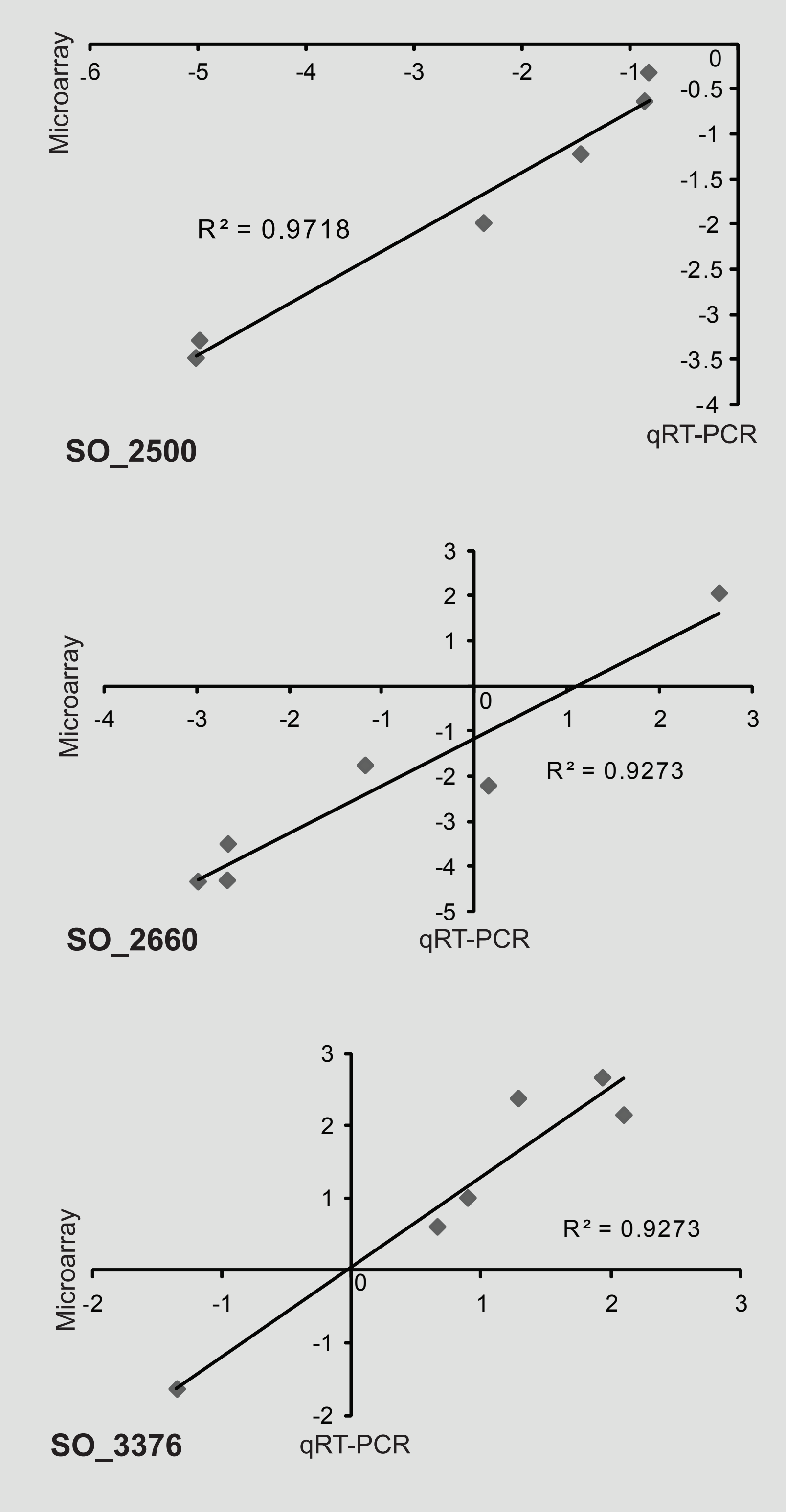

Figure S2: Validation of the transcriptome data by q-RT-PCR analyses. The diagrams are representing the log2-fold changes of the gene expression of SO_2500, SO_2660 and SO_3376 in surface-associated cells under hydrodynamic conditions (harvested after 0.25, 1, 2 and 4 hours) compared to planktonic culture. Plotted on the x-axis are the data values of the microarray analyses, on the y-axis the data values of the q-RT-PCR are shown. The coefficient of determination R^2 represents the statistical variance of the transcriptome data.
