# Supplementary material for: Transcriptome Analysis of Early Surface-Associated Growth of Shewanella oneidensis MR-1
Source: PLoS One. 2012 Jul 31;7(7):e42160. doi: 10.1371/journal.pone.0042160 (PMC3409153; doi:10.1371/journal.pone.0042160)
Supplement: Table S1 — Genes significantly upregulated after 60 minutes of attachment compared to 15 minutes of attachment. (PDF) [file pone.0042160.s003.pdf]

Table S1: Genes significantly upregulated after 60 minutes of attachment compared to 15 minutes of attachment.

Locus	Gene	logFC	Product	COG description
SO_0042	-	1.17	carbonic anhydrase	General function prediction only
SO_0046	-	1.07	hypothetical protein	Function unknown
SO_0055	-	1.06	hypothetical protein	General function prediction only
SO_0062	-	1.02	hypothetical protein	not in COGs
SO_0068	-	1.12	hypothetical protein	not in COGs
SO_0113	-	1.48	hypothetical protein	not in COGs
SO_0139	*ftn*	1.80	ferritin	Inorganic ion transport and metabolism
SO_0172	*gspJ*	1.30	general secretion pathway protein J	Intracellular trafficking, secretion, and vesicular transport
SO_0205	-	1.26	hypothetical protein	Cell wall/membrane/envelope biogenesis
SO_0215	*panK*	1.23	pantothenate kinase	Coenzyme transport and metabolism
SO_0258	-	1.95	hypothetical protein	not in COGs
SO_0285	-	1.18	type IV pilus biogenesis protein PilQ	Intracellular trafficking, secretion, and vesicular transport
SO_0322	-	1.54	hypothetical protein	Function unknown
SO_0341	-	1.33	sensory box protein	Signal transduction mechanisms
SO_0355	-	1.64	AMP-binding protein	Lipid transport and metabolism
SO_0429	-	1.32	M13 family peptidase	Posttranslational modification, protein turnover, chaperones
SO_0433	*rsd*	1.12	anti-RNA polymerase sigma 70 factor	Transcription
SO_0452	*trxC*	1.44	thioredoxin 2	Posttranslational modification, protein turnover, chaperones
SO_0496	-	1.31	hypothetical protein	Lipid transport and metabolism
SO_0501	-	1.28	hypothetical protein	not in COGs
SO_0522	*mscL*	1.03	large-conductance mechanosensitive channel	Cell wall/membrane/envelope biogenesis
SO_0523	-	1.27	LysR family transcriptional regulator	Transcription
SO_0542	-	1.14	hypothetical protein	Defense mechanisms
SO_0566	-	1.19	ABC 3 transport family protein	Inorganic ion transport and metabolism
SO_0630	*nosA*	1.51	TonB-dependent receptor	Inorganic ion transport and metabolism
SO_0640	-	1.24	alcohol dehydrogenase, zinc-containing	Energy metabolism
SO_0714	-	2.39	monoheme cytochrome c	Energy metabolism
SO_0725	*katG-1*	1.47	catalase/peroxidase HPI	Inorganic ion transport and metabolism
SO_0733	-	1.12	cold shock domain-contain protein	Transcription
SO_0797	-	1.24	hypothetical protein	Posttranslational modification, protein turnover, chaperones
SO_0798	-	1.93	hypothetical protein	Inorganic ion transport and metabolism
SO_0830	-	1.04	alkaline phosphatase	Inorganic ion transport and metabolism
SO_0858	-	2.46	sodium:alanine symporter family protein	Amino acid transport and metabolism
SO_0864	-	1.08	LuxR family transcriptional regulator	Signal transduction mechanisms
SO_0895	-	1.07	pirin family protein	General function prediction only
SO_0898	-	1.08	hypothetical protein	Function unknown
SO_0900	-	1.28	aldo/keto reductase family oxidoreductase	Energy metabolism
SO_0909	-	1.23	hypothetical protein	not in COGs
SO_0912	-	1.28	hypothetical protein	not in COGs
SO_0919	-	1.13	serine transporter, putative	Amino acid transport and metabolism
SO_0923	-	1.44	hypothetical protein	Posttranslational modification, protein turnover, chaperones
SO_0956	*ahpF*	2.38	alkyl hydroperoxide reductase, F subunit	Posttranslational modification, protein turnover, chaperones
SO_0958	*ahpC*	2.22	alkyl hydroperoxide reductase, C subunit	Posttranslational modification, protein turnover, chaperones
SO_0996	-	1.19	glyoxalase family protein	Amino acid transport and metabolism
SO_1014	*nuoI*	1.11	NADH dehydrogenase subunit I	Energy metabolism
SO_1020	*nuoB*	1.13	NADH dehydrogenase subunit B	Energy metabolism
SO_1066	-	1.48	extracellular nuclease	General function prediction only

SO_1070	*katB*	1.37	catalase	Inorganic ion transport and metabolism
SO_1075	-	1.80	hypothetical protein	not in COGs
SO_1076	-	2.55	hypothetical protein	not in COGs
SO_1152	-	1.04	hypothetical protein	not in COGs
SO_1188	-	1.98	hypothetical protein	Function unknown
SO_1189	-	1.55	hypothetical protein	Function unknown
SO_1190	-	1.48	hypothetical protein	Inorganic ion transport and metabolism
SO_1208	-	1.47	GGDEF domain-containing protein	Signal transduction mechanisms
SO_1271	-	1.01	polyamine ABC transporter, ATP-binding protein	Amino acid transport and metabolism
SO_1290	-	1.03	putative glycerol-3-phosphate acyltransferase PlsY	Function unknown
SO_1367	*pheA*	1.17	chorismate mutase/prephenate dehydratase	Amino acid transport and metabolism
SO_1383	-	1.09	DEAD-box ATP dependent DNA helicase	Replication, recombination and repair
SO_1482	-	5.02	TonB-dependent receptor, putative	Inorganic ion transport and metabolism
SO_1505	-	1.46	transporter, putative	Carbohydrate transport and metabolism
SO_1537	-	1.21	hypothetical protein	not in COGs
SO_1556	*rdgC*	1.01	recombination associated protein	Replication, recombination and repair
SO_1563	-	1.24	glutathione peroxidase, putative	Posttranslational modification, protein turnover, chaperones
SO_1571	-	1.03	hypothetical protein	Function unknown
SO_1579	-	1.29	hypothetical protein	Function unknown
SO_1580	-	2.42	TonB-dependent heme receptor	Inorganic ion transport and metabolism
SO_1606	-	1.64	metallo-beta-lactamase superfamily protein	Secondary metabolites biosynthesis, transport and catabolism
SO_1698	-	1.63	hypothetical protein	not in COGs
SO_1726	*phoU*	1.07	transcriptional regulator PhoU	Inorganic ion transport and metabolism
SO_1727	-	1.75	hypothetical protein	not in COGs
SO_1737	-	1.09	hypothetical protein	not in COGs
SO_1766	-	1.21	hypothetical protein	not in COGs
SO_1863	-	1.08	HU family DNA-binding protein	Replication, recombination and repair
SO_1864	-	1.39	hypothetical protein	not in COGs
SO_1865	-	1.18	ABC transporter, ATP-binding protein	Defense mechanisms
SO_1949	-	1.83	invasin domain-containing protein	not in COGs
SO_1955	-	2.28	hypothetical protein	not in COGs
SO_2001	*ushA*	1.77	bifunctional UDP-sugar hydrolase/5'-nucleotidase periplasmic precursor	Nucleotide transport and metabolism
SO_2039	-	2.13	hypothetical protein	Signal transduction mechanisms
SO_2178	*ccpA*	1.32	cytochrome c551 peroxidase	Inorganic ion transport and metabolism
SO_2216	-	1.35	sensory box protein	Signal transduction mechanisms
SO_2248	*sdaA*	1.05	L-serine dehydratase 1	Amino acid transport and metabolism
SO_2370	-	1.09	phage integrase family site specific recombinase	Replication, recombination and repair
SO_2411	*gyrA*	1.18	DNA gyrase, A subunit	Replication, recombination and repair
SO_2426	-	1.25	DNA-binding response regulator	Signal transduction mechanisms
SO_2486	*eda*	2.23	keto-hydroxyglutarate-aldolase/keto-deoxy-phosphogluconate aldolase	Carbohydrate transport and metabolism
SO_2487	*edd*	1.68	phosphogluconate dehydratase	Amino acid transport and metabolism
SO_2488	*pgl*	2.34	6-phosphogluconolactonase	Carbohydrate transport and metabolism
SO_2489	*zwf*	2.01	glucose-6-phosphate 1-dehydrogenase	Carbohydrate transport and metabolism
SO_2491	*pykA*	2.23	pyruvate kinase II	Carbohydrate transport and metabolism
SO_2560	*rnhA*	1.07	ribonuclease HI	Replication, recombination and repair
SO_2570	-	1.05	putative lipoprotein	Function unknown
SO_2737	*bioD*	1.23	dithiobiotin synthetase	Coenzyme transport and metabolism
SO_2740	*bioB*	1.28	biotin synthase	Coenzyme transport and metabolism
SO_2741	*bioA*	1.07	adenosylmethionine--8-amino-7-oxononanoate aminotransferase	Coenzyme transport and metabolism
SO_2763	-	1.26	hypothetical protein	Transcription
SO_2766	-	1.14	hypothetical protein	General function prediction only
SO_2772	-	1.02	cytosolic long-chain acyl-CoA thioester hydrolase family protein	Lipid transport and metabolism

SO_2828	-	1.09	hypothetical protein	not in COGs
SO_2843	-	1.01	exonuclease SbcC, putative	Replication, recombination and repair
SO_2844	-	1.35	exonuclease SbcD, putative	Replication, recombination and repair
SO_2848	-	2.09	hypothetical protein	not in COGs
SO_2866	*iciA*	1.02	chromosome replication initiation inhibitor protein	Transcription
SO_2888	-	2.43	hypothetical protein	not in COGs
SO_2900	-	1.89	hypothetical protein	not in COGs
SO_2907	-	1.56	TonB-dependent receptor domain-containing protein	Inorganic ion transport and metabolism
SO_2939	-	1.10	hypothetical protein	not in COGs
SO_2960	-	1.43	hypothetical protein	not in COGs
SO_2963	-	1.19	prophage LambdaSo, HK97 family major capsid protein	not in COGs
SO_2964	-	1.29	ClpP protease family protein	Posttranslational modification, protein turnover, chaperones
SO_2965	-	1.34	prophage LambdaSo, HK97 family portal protein	Function unknown
SO_2969	-	2.21	prophage LambdaSo, holin, putative	Defense mechanisms
SO_2970	-	1.81	hypothetical protein	Carbohydrate transport and metabolism
SO_2971	-	2.02	hypothetical protein	not in COGs
SO_2972	-	1.91	hypothetical protein	not in COGs
SO_2974	-	2.76	hypothetical protein	not in COGs
SO_3012	-	1.09	hypothetical protein	not in COGs
SO_3019	*trpE*	1.02	anthranilate synthase component I	Amino acid transport and metabolism
SO_3020	*trpG*	1.45	anthranilate synthase component II	Amino acid transport and metabolism
SO_3022	*trpC/F*	1.13	bifunctional indole-3-glycerol phosphate synthase/phosphoribosylanthranilate isomerase	Amino acid transport and metabolism
SO_3023	*trpB*	1.46	tryptophan synthase subunit beta	Amino acid transport and metabolism
SO_3024	*trpA*	1.77	tryptophan synthase subunit alpha	Amino acid transport and metabolism
SO_3025	-	1.89	hypothetical protein	General function prediction only
SO_3030	*alcA*	5.33	siderophore biosynthesis protein	Secondary metabolites biosynthesis, transport and catabolism
SO_3031	-	3.94	siderophore biosynthesis protein, putative	Translation, ribosomal structure and biogenesis
SO_3032	-	5.81	siderophore biosynthesis protein, putative	Secondary metabolites biosynthesis, transport and catabolism
SO_3033	-	5.61	ferric alcaligin siderophore receptor	Inorganic ion transport and metabolism
SO_3062	-	2.04	hypothetical protein	not in COGs
SO_3083	-	1.15	M16 family peptidase	Posttranslational modification, protein turnover, chaperones
SO_3101	-	2.37	hypothetical protein	Function unknown
SO_3102	-	1.91	AcrA/AcrE family protein	Cell wall/membrane/envelope biogenesis
SO_3142	*dcp-1*	1.36	peptidyl-dipeptidase Dcp	Amino acid transport and metabolism
SO_3244	*flgG*	1.08	flagellar basal body rod protein FlgG	Cell motility
SO_3259	-	1.07	hypothetical protein	Function unknown
SO_3263	-	1.08	3-oxoacyl-(acyl-carrier-protein) reductase, putative	Lipid transport and metabolism
SO_3265	-	1.61	hypothetical protein	Lipid transport and metabolism
SO_3266	-	1.50	hypothetical protein	Secondary metabolites biosynthesis, transport and catabolism
SO_3269	-	1.44	cytidyltransferase-like protein	Cell wall/membrane/envelope biogenesis
SO_3273	-	1.63	hypothetical protein	General function prediction only
SO_3326	-	1.40	hypothetical protein	Function unknown
SO_3335	-	1.03	hypothetical protein	Function unknown
SO_3343	-	1.42	hypothetical protein	not in COGs
SO_3344	-	1.57	hypothetical protein	not in COGs
SO_3361	-	1.59	hypothetical protein	Function unknown
SO_3369	-	1.09	hypothetical protein	Energy metabolism
SO_3370	-	1.19	hypothetical protein	Function unknown
SO_3371	-	1.36	cytochrome B561	Energy metabolism
SO_3387	-	1.79	hypothetical protein	not in COGs
SO_3391	-	1.31	ATP-dependent protease, putative	Posttranslational modification, protein turnover, chaperones
SO_3392	-	1.05	oxidoreductase, FMN-binding	Energy metabolism

SO_3402	-	1.63	hypothetical protein	Secondary metabolites biosynthesis, transport and catabolism
SO_3403	*yfiA-1*	1.21	ribosomal subunit interface protein	Translation, ribosomal structure and biogenesis
SO_3406	-	1.86	hypothetical protein	not in COGs
SO_3407	-	1.85	hypothetical protein	Function unknown
SO_3408	-	1.66	hypothetical protein	not in COGs
SO_3411	-	1.39	protease, putative	Cell wall/membrane/envelope biogenesis
SO_3413	*thrC*	1.65	threonine synthase	Amino acid transport and metabolism
SO_3547	*pgi*	1.67	glucose-6-phosphate isomerase	Carbohydrate transport and metabolism
SO_3560	-	1.53	M16 family peptidase	General function prediction only
SO_3665	-	2.00	ABC transporter, ATP-binding/permease protein, putative	Defense mechanisms
SO_3667	-	3.62	hypothetical protein	Inorganic ion transport and metabolism
SO_3668	-	3.64	hypothetical protein	Inorganic ion transport and metabolism
SO_3669	*hugA*	2.71	heme transport protein	Inorganic ion transport and metabolism
SO_3670	*tonB1*	3.51	TonB1 protein	Cell wall/membrane/envelope biogenesis
SO_3671	*exbB1*	5.70	TonB system transport protein ExbB1	Intracellular trafficking, secretion, and vesicular transport
SO_3672	*exbD1*	5.38	TonB system transport protein ExbD1	Intracellular trafficking, secretion, and vesicular transport
SO_3673	*hmuT*	4.04	hemin ABC transporter, periplasmic hemin-binding protein	Inorganic ion transport and metabolism
SO_3674	*hmuU*	2.33	hemin ABC transporter, permease protein	Inorganic ion transport and metabolism
SO_3675	*hmuV*	5.03	hemin importer ATP-binding subunit	Inorganic ion transport and metabolism
SO_3676	-	1.82	hypothetical protein	not in COGs
SO_3731	-	1.33	hypothetical protein	Cell wall/membrane/envelope biogenesis
SO_3758	-	1.04	hypothetical protein	General function prediction only
SO_3775	-	1.11	hypothetical protein	not in COGs
SO_3781	-	1.30	hypothetical protein	not in COGs
SO_3783	-	1.71	DEAD-box ATP dependent DNA helicase	Replication, recombination and repair
SO_3801	-	1.03	ABC transporter, permease protein	Defense mechanisms
SO_3879	-	1.07	hypothetical protein	not in COGs
SO_3911	-	1.37	hypothetical protein	not in COGs
SO_3913	-	4.18	putative hydroxylase	Function unknown
SO_3914	-	3.15	TonB-dependent receptor, putative	Inorganic ion transport and metabolism
SO_3974	-	1.06	hypothetical protein	not in COGs
SO_4010	-	2.19	hypothetical protein	not in COGs
SO_4011	-	1.17	hypothetical protein	not in COGs
SO_4017	-	1.03	Slt family transglycosylase	Cell wall/membrane/envelope biogenesis
SO_4022	-	1.34	M16 family peptidase	General function prediction only
SO_4068	-	2.10	hypothetical protein	not in COGs
SO_4077	-	4.70	TonB-dependent receptor, putative	Inorganic ion transport and metabolism
SO_4134	-	1.29	hypothetical protein	Function unknown
SO_4196	-	1.47	hypothetical protein	not in COGs
SO_4243	*rarD*	1.32	rarD protein	General function prediction only
SO_4275	-	1.11	hypothetical protein	not in COGs
SO_4282	*ktrB*	1.18	potassium uptake protein KtrB	Inorganic ion transport and metabolism
SO_4285	-	1.05	hypothetical protein	not in COGs
SO_4290	*pstA*	1.43	phosphate ABC transporter, permease protein	Inorganic ion transport and metabolism
SO_4291	*pstC*	1.63	phosphate ABC transporter, permease protein	Inorganic ion transport and metabolism
SO_4292	*pstS*	1.75	phosphate ABC transporter, periplasmic phosphate-binding protein	Inorganic ion transport and metabolism
SO_4344	*ilvA*	1.22	threonine dehydratase	Amino acid transport and metabolism
SO_4346	*ilvM*	1.50	acetolactate synthase 2 regulatory subunit	Function unknown
SO_4349	*ilvC*	1.63	ketol-acid reductoisomerase	Amino acid transport and metabolism
SO_4391	-	1.03	hypothetical protein	not in COGs
SO_4394	*pspE-2*	2.39	phage shock protein E	Inorganic ion transport and metabolism
SO_4395	-	1.47	hypothetical protein	not in COGs

SO_4419	-	1.02	hypothetical protein	Amino acid transport and metabolism
SO_4443	-	1.47	hypothetical protein	not in COGs
SO_4445	-	1.13	response regulator/sensor histidine kinase	Signal transduction mechanisms
SO_4454	-	2.50	methyl-accepting chemotaxis protein	Cell motility
SO_4469	-	1.19	alcohol dehydrogenase, iron-containing	Energy metabolism
SO_4472	*ntrC*	1.50	nitrogen regulation protein NR(I)	Signal transduction mechanisms
SO_4477	*cpxR*	1.31	transcriptional regulatory protein CpxR	Signal transduction mechanisms
SO_4516	*viuA*	3.60	ferric vibriobactin receptor	Inorganic ion transport and metabolism
SO_4523	*irgA*	1.80	enterobactin receptor protein	Inorganic ion transport and metabolism
SO_4524	-	1.17	LysR family transcriptional regulator	Transcription
SO_4535	-	1.14	hypothetical protein	Amino acid transport and metabolism
SO_4618	-	1.46	prolyl oligopeptidase family protein	Amino acid transport and metabolism
SO_4663	-	3.26	hypothetical protein	not in COGs
SO_4680	-	1.26	hypothetical protein	Cell wall/membrane/envelope biogenesis
SO_4693	-	1.39	multidrug resistance protein AcrA/AcrE family	Cell wall/membrane/envelope biogenesis
SO_4715	-	1.58	hypothetical protein	Function unknown
SO_4716	-	1.15	acetyltransferase	Transcription
SO_4740	-	1.47	hypothetical protein	Function unknown
SO_4743	-	1.76	TonB-dependent receptor, putative	Inorganic ion transport and metabolism
SO_A0003	-	2.37	type II restriction endonuclease, putative	not in COGs
SO_A0011	-	1.04	hypothetical protein	not in COGs
SO_A0030	-	1.05	hypothetical protein	not in COGs
SO_A0048	-	1.32	prolyl oligopeptidase family protein	Amino acid transport and metabolism
SO_A0049	-	1.30	toxin secretion ABC transporter, ATP-binding subunit/permease protein, putative	Defense mechanisms
SO_A0051	-	1.13	hypothetical protein	not in COGs
SO_A0177	-	1.76	bacteriocin-like peptide	not in COGs
SO_A0178	-	1.31	bacteriocin-like peptide	not in COGs
