# Supplementary material for: Transcriptome Analysis of Early Surface-Associated Growth of Shewanella oneidensis MR-1
Source: PLoS One. 2012 Jul 31;7(7):e42160. doi: 10.1371/journal.pone.0042160 (PMC3409153; doi:10.1371/journal.pone.0042160)
Supplement: Table S2 — Genes significantly downregulated after 60 minutes of attachment compared to 15 minutes of attachment. (PDF) [file pone.0042160.s004.pdf]

Table S2: Genes significantly downregulated after 60 minutes of attachment compared to 15 minutes of attachment.

Locus	Gene	logFC	Product	COG description
SO_0020	*fadA*	-1.83	3-ketoacyl-CoA thiolase	Lipid transport and metabolism
SO_0021	*fadB*	-1.73	multifunctional fatty acid oxidation complex subunit alpha	Lipid transport and metabolism
SO_0085	-	-1.40	hypothetical protein	not in COGs
SO_0094	-	-1.03	hypothetical protein	not in COGs
SO_0101	*fdnG*	-2.03	selenium-containing formate dehydrogenase, nitrate inducible, alpha subunit	Energy metabolism
SO_0102	*fdnH*	-1.89	formate dehydrogenase, nitrate-inducible, iron-sulfur subunit	Energy metabolism
SO_0103	*fdnI*	-2.13	formate dehydrogenase, nitrate-inducible, cytochrome b556 subunit	Energy production and conversion
SO_0104	*fdhE*	-1.38	fdhE protein	Posttranslational modification, protein turnover, chaperones
SO_0105	*selA*	-3.44	selenocysteine synthase	Amino acid transport and metabolism
SO_0106	*selB*	-2.79	selenocysteine-specific translation elongation factor	Translation, ribosomal structure and biogenesis
SO_0107	*fdhD*	-2.90	formate dehydrogenase accessory protein FdhD	Energy metabolism
SO_0108	-	-2.51	putative inner membrane protein	General function prediction only
SO_0109	-	-1.94	hypothetical protein	Posttranslational modification, protein turnover, chaperones
SO_0148	-	-1.24	hypothetical protein	Translation, ribosomal structure and biogenesis
SO_0150	-	-2.62	putative lipoprotein	Function unknown
SO_0151	-	-2.05	SAM-dependent methyltransferase	Secondary metabolites biosynthesis, transport and catabolism
SO_0154	-	-1.27	hypothetical protein	Function unknown
SO_0275	*argC*	-1.34	N-acetyl-gamma-glutamyl-phosphate reductase	Amino acid transport and metabolism
SO_0336	-	-1.62	Na(+)/H(+) antiporter	Energy metabolism
SO_0343	*acnA*	-1.00	aconitate hydratase	Energy metabolism
SO_0344	*prpC*	-1.61	methylcitrate synthase	Energy metabolism
SO_0345	*prpB*	-1.58	2-methylisocitrate lyase	Carbohydrate transport and metabolism
SO_0346	-	-1.37	GntR family transcriptional regulator	Transcription
SO_0388	-	-1.02	phage integrase family site specific recombinase	Replication, recombination and repair
SO_0396	*frdC*	-2.31	fumarate reductase cytochrome b-556 subunit	Energy production and conversion
SO_0439	-	-1.59	hypothetical protein	not in COGs
SO_0441	*purD*	-1.51	phosphoribosylamine--glycine ligase	Nucleotide transport and metabolism
SO_0442	*purH*	-1.80	bifunctional phosphoribosylaminoimidazolecarboxamide formyltransferase	Nucleotide transport and metabolism
SO_0455	-	-2.43	transporter	General function prediction only
SO_0456	-	-2.10	immunogenic-related protein	General function prediction only
SO_0477	*nrfF*	-1.59	cytochrome c-type biogenesis protein NrfF precursor	Posttranslational modification, protein turnover, chaperones
SO_0484	*nrfD*	-1.41	formate-dependent nitrite reductase, nrfD protein	Inorganic ion transport and metabolism
SO_0488	*nosY*	-1.13	copper ABC transporter, permease protein	General function prediction only
SO_0524	-	-1.11	HlyD family secretion protein	Defense mechanisms
SO_0525	-	-1.54	drug resistance transporter, EmrB/QacA family protein	Carbohydrate transport and metabolism
SO_0532	*arsR*	-1.44	arsenical resistence operon repressor	Transcription
SO_0533	-	-1.57	arsenate reductase, putative	Signal transduction mechanisms
SO_0535	-	-1.15	hypothetical protein	General function prediction only
SO_0538	*gapA-1*	-1.43	glyceraldehyde-3-phosphate dehydrogenase	Carbohydrate transport and metabolism
SO_0543	-	-2.38	hypothetical protein	not in COGs
SO_0544	-	-1.15	sensory box histidine kinase	Signal transduction mechanisms
SO_0572	-	-1.02	enoyl-CoA hydratase	Lipid transport and metabolism
SO_0581	-	-1.48	hypothetical protein	not in COGs
SO_0651	-	-1.27	hypothetical protein	Function unknown
SO_0701	-	-1.04	LysR family transcriptional regulator	Transcription
SO_0727	-	-1.64	hypothetical protein	General function prediction only
SO_0728	-	-1.44	hypothetical protein	Function unknown

SO_0748	-	-2.86	hypothetical protein	not in COGs
SO_0760	*amt*	-2.58	ammonium transporter	Inorganic ion transport and metabolism
SO_0781	*gcvP*	-1.25	glycine dehydrogenase	Amino acid transport and metabolism
SO_0837	-	-1.09	beta-lactamase, putative	Defense mechanisms
SO_0843	-	-1.29	LysR family transcriptional regulator	Transcription
SO_0844	-	-1.43	hypothetical protein	not in COGs
SO_0845	*napB*	-2.57	cytochrome c-type protein NapB	Energy metabolism
SO_0846	*napH*	-2.25	quinol dehydrogenase membrane component	Energy metabolism
SO_0847	*napG*	-2.40	quinol dehydrogenase periplasmic component	Energy metabolism
SO_0848	*napA*	-2.43	nitrate reductase	Energy metabolism
SO_0849	*napD*	-3.04	napD protein	Inorganic ion transport and metabolism
SO_0850	-	-1.87	hypothetical protein	not in COGs
SO_0859	-	-1.64	sensory box histidine kinase/response regulator	Signal transduction mechanisms
SO_0940	-	-1.35	transcriptional regulator-related protein	Transcription
SO_0945	-	-1.33	AcrB/AcrD/AcrF family protein	Defense mechanisms
SO_0946	-	-1.63	hypothetical protein	Cell wall/membrane/envelope biogenesis
SO_0948	-	-1.15	hypothetical protein	General function prediction only
SO_0970	-	-1.00	fumarate reductase flavoprotein subunit precursor	Energy metabolism
SO_0988	-	-1.86	formate dehydrogenase, alpha subunit	Energy metabolism
SO_1047	-	-2.05	hypothetical protein	General function prediction only
SO_1048	-	-1.98	hypothetical protein	Cell wall/membrane/envelope biogenesis
SO_1049	-	-1.43	acetyltransferase	Translation, ribosomal structure and biogenesis
SO_1077	-	-1.16	hypothetical protein	not in COGs
SO_1120	-	-1.98	xanthine/uracil permease family protein	General function prediction only
SO_1141	*carA*	-1.29	carbamoyl phosphate synthase small subunit	Amino acid transport and metabolism
SO_1213	-	-1.27	TatD family hydrolase	Replication, recombination and repair
SO_1232	*torA*	-1.00	trimethylamine-N-oxide reductase	Energy metabolism
SO_1236	-	-1.12	xanthine/uracil permease family protein	General function prediction only
SO_1251	-	-1.03	ferredoxin, 4Fe-4S	Energy metabolism
SO_1267	-	-1.79	glutamine amidotransferase	General function prediction only
SO_1301	*pyrB*	-1.38	aspartate carbamoyltransferase	Nucleotide transport and metabolism
SO_1311	-	-1.33	transcriptional regulator	Transcription
SO_1326	-	-1.26	hypothetical protein	General function prediction only
SO_1363	*hcp*	-1.16	hydroxylamine reductase	Energy metabolism
SO_1399	-	-1.00	hypothetical protein	not in COGs
SO_1405	-	-2.77	transglutaminase family protein	Amino acid transport and metabolism
SO_1406	-	-1.95	mercuric transport protein MerT, putative	not in COGs
SO_1414	-	-2.54	flavocytochrome c flavin subunit, putative	Energy metabolism
SO_1415	-	-1.41	TetR family transcriptional regulator	Transcription
SO_1427	-	-3.22	decaheme cytochrome c	Energy metabolism
SO_1432	-	-1.16	hypothetical protein	not in COGs
SO_1449	-	-2.88	hypothetical protein	not in COGs
SO_1455	-	-1.38	RadC family DNA repair protein	Replication, recombination and repair
SO_1511	-	-1.07	IS91 family transposase	not in COGs
SO_1513	-	-1.33	hypothetical protein	Nucleotide transport and metabolism
SO_1532	-	-1.38	hypothetical protein	not in COGs
SO_1612	-	-1.28	hypothetical protein	not in COGs
SO_1655	*cysQ-2*	-1.23	cysQ protein	Inorganic ion transport and metabolism
SO_1663	*napF*	-1.14	ferredoxin-type protein NapF	Energy metabolism
SO_1677	*atoB*	-2.05	acetyl-CoA acetyltransferase	Lipid transport and metabolism
SO_1678	*mmsA*	-1.94	methylmalonate-semialdehyde dehydrogenase	Energy metabolism
SO_1679	-	-2.31	acyl-CoA dehydrogenase family protein	Lipid transport and metabolism

SO_1680	-	-2.30	enoyl-CoA hydratase	Lipid transport and metabolism
SO_1681	-	-1.78	enoyl-CoA hydratase/isomerase family protein	Lipid transport and metabolism
SO_1694	-	-3.09	FAD-binding protein	Energy metabolism
SO_1695	-	-4.39	sensory box/GGDEF family protein	Signal transduction mechanisms
SO_1697	-	-3.32	hypothetical protein	Replication, recombination and repair
SO_1707	-	-1.69	ABC transporter, permease protein, putative	Defense mechanisms
SO_1776	*mtrB*	-1.24	outer membrane protein precursor MtrB	not in COGs
SO_1778	*omcB*	-1.08	decaheme cytochrome c	not in COGs
SO_1787	-	-1.63	hypothetical protein	not in COGs
SO_1807	*pspA*	-1.25	phage shock protein A	Transcription
SO_1809	*pspC*	-1.14	phage shock protein C	Transcription
SO_1810	-	-1.44	ATPase	General function prediction only
SO_1812	*mdeA*	-1.27	methionine gamma-lyase	Amino acid transport and metabolism
SO_1813	-	-1.64	DNA-binding protein, putative	Replication, recombination and repair
SO_1834	-	-1.10	acetyltransferase	Translation, ribosomal structure and biogenesis
SO_1855	*rmf*	-1.06	ribosome modulation factor	Translation, ribosomal structure and biogenesis
SO_1891	-	-1.12	3-oxoadipate CoA-succinyl transferase, beta subunit	Lipid transport and metabolism
SO_1892	*atoD*	-1.78	acetate CoA-transferase, subunit A	Lipid transport and metabolism
SO_1893	*mvaB*	-1.32	hydroxymethylglutaryl-CoA lyase	Amino acid transport and metabolism
SO_1894	-	-1.80	acetyl-CoA carboxylase, biotin carboxylase, putative	Lipid transport and metabolism
SO_1895	-	-1.14	enoyl-CoA hydratase/isomerase family protein	Lipid transport and metabolism
SO_1896	*pccB-1*	-1.68	3-methylcrotonyl CoA carboxylase, beta subunit	Lipid transport and metabolism
SO_1897	*ivd*	-2.16	isovaleryl-CoA dehydrogenase	Lipid transport and metabolism
SO_1898	-	-1.34	transcriptional regulator, putative	Transcription
SO_1908	-	-1.01	hypothetical protein	not in COGs
SO_1915	-	-1.07	serine protease	Posttranslational modification, protein turnover, chaperones
SO_1917	-	-1.44	multidrug resistance protein, putative	Carbohydrate transport and metabolism
SO_1924	-	-1.70	AcrB/AcrD/AcrF family protein	Defense mechanisms
SO_1925	-	-1.69	HlyD family secretion protein	Cell wall/membrane/envelope biogenesis
SO_1963	-	-1.51	hypothetical protein	Secondary metabolites biosynthesis, transport and catabolism
SO_1964	-	-5.32	hypothetical protein	not in COGs
SO_1976	-	-1.73	alpha/beta fold family hydrolase	General function prediction only
SO_1977	-	-1.12	hypothetical protein	Function unknown
SO_1987	-	-1.36	ATP-dependent protease La	General function prediction only
SO_1988	-	-1.72	methyltransferase	Secondary metabolites biosynthesis, transport and catabolism
SO_2005	-	-1.06	dksA-type zinc finger protein	Signal transduction mechanisms
SO_2019	*hemH-1*	-1.58	ferrochelatase	Coenzyme transport and metabolism
SO_2035	-	-1.34	transposase, putative	not in COGs
SO_2036	-	-1.18	phage integrase family site specific recombinase	Replication, recombination and repair
SO_2096	-	-1.71	hydrogenase expression/formation protein	Energy metabolism
SO_2097	*hydC*	-1.22	quinone-reactive Ni/Fe hydrogenase, cytochrome b subunit	Energy metabolism
SO_2098	*hyaB*	-1.87	quinone-reactive Ni/Fe hydrogenase, large subunit	Energy metabolism
SO_2102	-	-1.60	hypothetical protein	not in COGs
SO_2109	-	-1.55	hypothetical protein	Function unknown
SO_2121	*cheA*	-1.39	chemotaxis protein CheA	Cell motility
SO_2122	*cheW-1*	-1.63	purine-binding chemotaxis protein CheW	Cell motility
SO_2137	-	-1.45	hypothetical protein	Intracellular trafficking, secretion, and vesicular transport
SO_2143	-	-1.55	hypothetical protein	General function prediction only
SO_2144	-	-1.33	hypothetical protein	Energy metabolism
SO_2156	-	-1.27	alpha amylase family protein	Carbohydrate transport and metabolism
SO_2165	-	-1.19	IS91 family transposase	not in COGs
SO_2174	-	-1.58	cyclic nucleotide phosphodiesterase, putative	Signal transduction mechanisms

SO_2194	-	-4.07	OmpA family protein	Cell wall/membrane/envelope biogenesis
SO_2195	-	-2.13	inter-alpha-trypsin inhibitor domain-containing protein	General function prediction only
SO_2212	-	-1.65	IS91 family transposase	not in COGs
SO_2281	-	-1.37	alkaline phosphatase	Function unknown
SO_2347	*gapA-3*	-2.88	glyceraldehyde-3-phosphate dehydrogenase	Carbohydrate transport and metabolism
SO_2366	-	-1.48	response regulator	Transcription
SO_2389	*emrD*	-1.77	multidrug resistance protein D	Carbohydrate transport and metabolism
SO_2393	-	-1.20	hypothetical protein	not in COGs
SO_2395	-	-1.73	acyl-CoA dehydrogenase family protein	Lipid transport and metabolism
SO_2408	-	-1.07	radical activating enzyme	Posttranslational modification, protein turnover, chaperones
SO_2419	-	-2.13	2,4-dienoyl-CoA reductase, putative	General function prediction only
SO_2441	*thiG*	-1.94	thiazole synthase	Coenzyme transport and metabolism
SO_2442	*thiS*	-2.21	thiS protein, putative	Coenzyme transport and metabolism
SO_2444	*thiDE*	-1.61	phosphomethylpyrimidine kinase/thiamin-phosphate pyrophosphorylase, putative	Coenzyme transport and metabolism
SO_2492	-	-1.18	acyl-CoA dehydrogenase	Lipid transport and metabolism
SO_2493	-	-2.02	TetR family transcriptional regulator	Transcription
SO_2498	-	-2.11	sensory box protein	Signal transduction mechanisms
SO_2499	-	-2.01	hypothetical protein	Function unknown
SO_2500	-	-2.39	hypothetical protein	not in COGs
SO_2514	*nth*	-1.05	endonuclease III	Replication, recombination and repair
SO_2519	-	-1.36	AraC family transcriptional regulator	Transcription
SO_2527	-	-1.38	IS91 family transposase	not in COGs
SO_2536	*fadE*	-3.42	acyl-CoA dehydrogenase	Lipid transport and metabolism
SO_2547	-	-1.14	response regulator	Signal transduction mechanisms
SO_2585	-	-1.15	hypothetical protein	not in COGs
SO_2599	-	-1.23	hypothetical protein	not in COGs
SO_2644	*ppsA*	-1.15	phosphoenolpyruvate synthase	Carbohydrate transport and metabolism
SO_2645	-	-1.39	hypothetical protein	Function unknown
SO_2653	-	-2.60	Ner family transcriptional regulator	Transcription
SO_2654	-	-3.01	transposase, putative	Replication, recombination and repair
SO_2656	-	-2.86	hypothetical protein	not in COGs
SO_2657	-	-2.32	hypothetical protein	not in COGs
SO_2658	-	-1.72	hypothetical protein	not in COGs
SO_2659	-	-2.63	hypothetical protein	not in COGs
SO_2660	-	-1.96	hypothetical protein	not in COGs
SO_2661	-	-1.66	hypothetical protein	not in COGs
SO_2663	-	-1.91	hypothetical protein	not in COGs
SO_2664	-	-1.82	hypothetical protein	not in COGs
SO_2665	-	-3.17	hypothetical protein	not in COGs
SO_2666	-	-2.14	hypothetical protein	not in COGs
SO_2668	-	-1.00	prophage MuSo2, positive regulator of late transcription, putative	Function unknown
SO_2671	-	-1.48	hypothetical protein	General function prediction only
SO_2676	-	-1.68	hypothetical protein	Transcription
SO_2684	-	-2.15	prophage MuSo2, protein Gp32, putative	General function prediction only
SO_2685	-	-1.85	prophage MuSo2, major head subunit, putative	General function prediction only
SO_2686	-	-1.48	hypothetical protein	not in COGs
SO_2687	-	-1.31	hypothetical protein	not in COGs
SO_2688	-	-1.65	hypothetical protein	not in COGs
SO_2690	-	-1.15	prophage MuSo2, virion morphogenesis protein, putative	General function prediction only
SO_2693	-	-1.19	prophage MuSo2, tail sheath protein, putative	General function prediction only
SO_2695	-	-1.79	hypothetical protein	Function unknown
SO_2700	-	-1.55	prophage MuSo2, baseplate assembly protein V	Function unknown

SO_2701	-	-1.39	hypothetical protein	Function unknown
SO_2703	-	-1.66	hypothetical protein	Function unknown
SO_2712	-	-1.69	hypothetical protein	Cell wall/membrane/envelope biogenesis
SO_2713	-	-1.48	transporter, putative	Coenzyme transport and metabolism
SO_2714	-	-1.77	hypothetical protein	not in COGs
SO_2728	*htpX*	-1.04	heat shock protein HtpX	Posttranslational modification, protein turnover, chaperones
SO_2743	*acs*	-2.22	acetyl-CoA synthetase	Lipid transport and metabolism
SO_2761	*purN*	-1.07	phosphoribosylglycinamide formyltransferase	Nucleotide transport and metabolism
SO_2768	-	-1.00	acyl-CoA dehydrogenase family protein	Lipid transport and metabolism
SO_2805	-	-1.46	hypothetical protein	Inorganic ion transport and metabolism
SO_2821	-	-1.47	hypothetical protein	not in COGs
SO_2833	*nrdG*	-1.43	anaerobic ribonucleotide reductase-activating protein	Posttranslational modification, protein turnover, chaperones
SO_2857	-	-2.13	sodium/solute symporter family protein	General function prediction only
SO_2858	-	-2.26	hypothetical protein	Function unknown
SO_2905	-	-1.16	O-methyltransferase, putative	Secondary metabolites biosynthesis, transport and catabolism
SO_2911	-	-1.33	formate transporter, putative	Inorganic ion transport and metabolism
SO_2913	*pflA*	-1.37	pyruvate formate lyase-activating enzyme 1	Posttranslational modification, protein turnover, chaperones
SO_2918	-	-3.62	hypothetical protein	not in COGs
SO_2976	-	-1.75	hypothetical protein	not in COGs
SO_3060	-	-1.39	outer membrane porin, putative	Cell wall/membrane/envelope biogenesis
SO_3081	-	-1.00	hypothetical protein	Function unknown
SO_3087	-	-1.33	hypothetical protein	not in COGs
SO_3088	*fadJ*	-1.41	multifunctional fatty acid oxidation complex subunit alpha	Lipid transport and metabolism
SO_3089	*fadI*	-2.25	3-ketoacyl-CoA thiolase	Lipid transport and metabolism
SO_3109	-	-1.20	hypothetical protein	not in COGs
SO_3147	-	-1.55	hypothetical protein	not in COGs
SO_3228	*fliF*	-1.36	flagellar MS-ring protein	Cell motility
SO_3256	-	-1.28	hypothetical protein	Function unknown
SO_3297	-	-1.03	LysR family transcriptional regulator	Transcription
SO_3299	-	-1.40	Pal/histidase family protein	Amino acid transport and metabolism
SO_3300	-	-2.66	cytochrome c	Posttranslational modification, protein turnover, chaperones
SO_3318	-	-1.00	LysR family transcriptional regulator	Transcription
SO_3364	-	-1.41	hypothetical protein	not in COGs
SO_3384	*phrB*	-1.25	deoxyribodipyrimidine photolyase	Replication, recombination and repair
SO_3385	-	-3.20	MerR family transcriptional regulator	Transcription
SO_3386	-	-2.80	hypothetical protein	Function unknown
SO_3390	-	-1.27	hypothetical protein	not in COGs
SO_3492	*mexF*	-1.45	RND multidrug efflux transporter MexF	Defense mechanisms
SO_3493	*mexE*	-2.42	RND multidrug efflux membrane fusion protein MexE	Cell wall/membrane/envelope biogenesis
SO_3554	*purE*	-1.42	phosphoribosylaminoimidazole carboxylase, catalytic subunit	Nucleotide transport and metabolism
SO_3555	*purK*	-1.08	phosphoribosylaminoimidazole carboxylase ATPase subunit	Nucleotide transport and metabolism
SO_3603	-	-1.25	HlyD family secretion protein	Defense mechanisms
SO_3608	-	-1.08	serine/threonine protein kinase	General function prediction only
SO_3613	*purT*	-3.15	phosphoribosylglycinamide formyltransferase 2	Nucleotide transport and metabolism
SO_3641	*apaH*	-1.46	diadenosine tetraphosphatase	Signal transduction mechanisms
SO_3642	-	-1.07	methyl-accepting chemotaxis protein	Cell motility
SO_3645	-	-1.63	hypothetical protein	not in COGs
SO_3705	-	-3.10	5-methylthioadenosine nucleosidase/S-adenosylhomocysteine nucleosidase, putative	Nucleotide transport and metabolism
SO_3706	-	-2.69	NupC family protein	Nucleotide transport and metabolism
SO_3713	-	-1.08	hypothetical protein	not in COGs
SO_3714	-	-1.05	sugar-binding protein, putative	Carbohydrate transport and metabolism

SO_3725	-	-1.84	hypothetical protein	not in COGs
SO_3726	*cysN*	-1.62	sulfate adenylyltransferase subunit 1	Inorganic ion transport and metabolism
SO_3727	*cysD*	-2.29	sulfate adenylyltransferase subunit 2	Amino acid transport and metabolism
SO_3728	*cobA*	-3.32	uroporphyrin-III C-methyltransferase	Coenzyme transport and metabolism
SO_3737	*cysI*	-1.09	sulfite reductase subunit beta	Inorganic ion transport and metabolism
SO_3738	*cysJ*	-1.95	sulfite reductase (NADPH) flavoprotein alpha-component	Inorganic ion transport and metabolism
SO_3776	-	-1.19	hypothetical protein	Function unknown
SO_3860	-	-1.51	hypothetical protein	Amino acid transport and metabolism
SO_3874	-	-1.28	LysR family transcriptional regulator	Transcription
SO_3884	-	-1.91	phage integrase family site specific recombinase	Replication, recombination and repair
SO_3910	-	-1.24	hypothetical protein	not in COGs
SO_3920	*hydA*	-1.26	periplasmic Fe hydrogenase, large subunit	General function prediction only
SO_3922	-	-1.71	formate dehydrogenase, putative	Energy metabolism
SO_3980	*nrfA*	-1.73	cytochrome c nitrite reductase	Inorganic ion transport and metabolism
SO_3985	-	-1.56	hypothetical protein	General function prediction only
SO_3986	*lysC*	-1.65	aspartate kinase III	Amino acid transport and metabolism
SO_4018	-	-1.08	hypothetical protein	not in COGs
SO_4040	-	-1.55	integral membrane domain-containing protein	Carbohydrate transport and metabolism
SO_4066	*hemH*	-1.92	phosphoribosylaminoimidazole-succinocarboxamide synthase	Nucleotide transport and metabolism
SO_4131	-	-2.72	hypothetical protein	Cell wall/membrane/envelope biogenesis
SO_4145	-	-2.10	hypothetical protein	Signal transduction mechanisms
SO_4168	-	-1.62	hypothetical protein	General function prediction only
SO_4169	-	-1.59	hypothetical protein	General function prediction only
SO_4209	-	-1.51	hypothetical protein	Replication, recombination and repair
SO_4229	-	-2.46	hypothetical protein	not in COGs
SO_4244	-	-1.24	hypothetical protein	not in COGs
SO_4322	-	-1.00	hypothetical protein	Function unknown
SO_4354	-	-1.04	Fe-S-cluster oxidoreductase	General function prediction only
SO_4379	-	-1.28	hypothetical protein	not in COGs
SO_4404	-	-1.36	iron-sulfur cluster-binding protein	Energy metabolism
SO_4466	-	-1.07	methyl-accepting chemotaxis protein	Cell motility
SO_4480	*aldA*	-1.96	aldehyde dehydrogenase	Energy metabolism
SO_4483	-	-1.62	cytochrome b, putative	Energy metabolism
SO_4504	-	-1.25	hypothetical protein	not in COGs
SO_4512	-	-1.38	hypothetical protein	not in COGs
SO_4513	-	-1.59	formate dehydrogenase, alpha subunit	**Energy metabolism**
SO_4514	*fdhB-2*	-1.44	formate dehydrogenase, iron-sulfur subunit	Energy metabolism
SO_4515	-	-1.16	formate dehydrogenase, C subunit, putative	Energy metabolism
SO_4539	-	-1.58	serine protease	Posttranslational modification, protein turnover, chaperones
SO_4598	-	-1.52	CzcA family heavy metal efflux protein	Inorganic ion transport and metabolism
SO_4606	-	-3.17	cytochrome c oxidase, subunit II	Energy metabolism
SO_4624	-	-1.19	LuxR family transcriptional regulator	Transcription
SO_4625	*comF*	-2.34	competence protein ComF	General function prediction only
SO_4636	-	-1.63	putative lipoprotein	not in COGs
SO_4650	-	-1.32	hypothetical protein	General function prediction only
SO_4651	-	-2.29	hypothetical protein	Function unknown
SO_4652	*sbp*	-1.72	sulfate ABC transporter, periplasmic sulfate-binding protein	Inorganic ion transport and metabolism
SO_4653	*cysT-2*	-2.11	sulfate ABC transporter, permease protein	Posttranslational modification, protein turnover, chaperones
SO_4654	*cysW-2*	-1.14	sulfate ABC transporter, permease protein	Inorganic ion transport and metabolism
SO_4688	-	-1.22	glycosyl transferase, group 2 family protein	Cell wall/membrane/envelope biogenesis
SO_4689	-	-1.20	hypothetical protein	Function unknown
SO_4709	-	-1.63	hypothetical protein	not in COGs

SO_4721	-	-1.02	ABC transporter, ATP-binding protein	Inorganic ion transport and metabolism
SO_4722	*mobA*	-1.11	molybdopterin-guanine dinucleotide biosynthesis protein	Coenzyme transport and metabolism
SO_4723	-	-1.52	molybdopterin biosynthesis MoeA protein, putative	Coenzyme transport and metabolism
SO_4724	-	-1.24	molybdenum cofactor biosynthesis protein A	Coenzyme transport and metabolism
SO_4732	-	-1.06	hypothetical protein	Function unknown
SO_A0012	*rulB*	-1.01	SOS mutagenesis protein RulB	Replication, recombination and repair
SO_A0013	*rulA*	-1.55	SOS mutagenesis protein RulA	Transcription
SO_A0014	-	-1.76	hypothetical protein	not in COGs
SO_A0026	-	-1.21	resolvase family site-specific recombinase	Replication, recombination and repair
SO_A0031	-	-1.03	ParB family partitioning protein	Transcription
SO_A0036	-	-1.45	HicB-related protein	Function unknown
SO_A0040	*pemI*	-1.46	antitoxin module of toxin-antitoxin system	not in COGs
SO_A0058	-	-1.55	hypothetical protein	not in COGs
SO_A0060	-	-1.06	acetyltransferase	Transcription
SO_A0067	-	-1.22	hypothetical protein	not in COGs
SO_A0068	-	-1.44	hypothetical protein	Transcription
SO_A0070	-	-1.68	hypothetical protein	Amino acid transport and metabolism
SO_A0127	-	-1.27	hypothetical protein	not in COGs
SO_A0147	-	-1.06	hypothetical protein	not in COGs
SO_A0150	-	-1.18	hypothetical protein	not in COGs
SO_A0153	-	-2.18	CzcA family heavy metal efflux protein	Inorganic ion transport and metabolism
SO_A0154	-	-2.91	heavy metal efflux protein, putative	Cell wall/membrane/envelope biogenesis
SO_A0155	-	-1.90	hypothetical protein	not in COGs
SO_A0168	-	-1.04	ISSo9, nucleotidyltransferase domain-containing protein	General function prediction only
SO_A0173	-	-1.72	hypothetical protein	not in COGs
SO_A0183	-	-1.32	hypothetical protein	not in COGs
SO_A0184	-	-1.11	hypothetical protein	not in COGs
