# Supplementary material for: Transcriptome Analysis of Early Surface-Associated Growth of Shewanella oneidensis MR-1
Source: PLoS One. 2012 Jul 31;7(7):e42160. doi: 10.1371/journal.pone.0042160 (PMC3409153; doi:10.1371/journal.pone.0042160)
Supplement: Table S3 — Differentially regulated genes potentially related to cell lysis and eDNA turnover. (PDF) [file pone.0042160.s005.pdf]

Table S3: Differentially regulated genes potentially related to cell lysis and eDNA turnover

Locus	Product	log_2 ratio
SO_1047	hypothetical protein	-2.05
SO_1048	hypothetical protein	-1.98
SO_1066	extracellular nuclease	1.48
**MuSo2**		
SO_2653	Ner family transcriptional regulator	-2.60
SO_2654	transposase, putative	-3.01
SO_2656	hypothetical protein	-2.86
SO_2657	hypothetical protein	-2.32
SO_2658	hypothetical protein	-1.72
SO_2659	hypothetical protein	-2.63
SO_2660	hypothetical protein	-1.96
SO_2661	hypothetical protein	-1.66
SO_2663	hypothetical protein	-1.91
SO_2664	hypothetical protein	-1.82
SO_2665	hypothetical protein	-3.17
SO_2666	hypothetical protein	-2.14
SO_2668	prophage MuSo2, positive regulator of late transcription, putative	-1.00
SO_2671	hypothetical protein	-1.48
SO_2676	hypothetical protein	-1.68
SO_2684	prophage MuSo2, protein Gp32, putative	-2.15
SO_2685	prophage MuSo2, major head subunit, putative	-1.85
SO_2686	hypothetical protein	-1.48
SO_2687	hypothetical protein	-1.31
SO_2688	hypothetical protein	-1.65
SO_2690	prophage MuSo2, virion morphogenesis protein, putative	-1.15
SO_2693	prophage MuSo2, tail sheath protein, putative	-1.19
SO_2695	hypothetical protein	-1.79
SO_2700	prophage MuSo2, baseplate assembly protein V	-1.55
SO_2701	hypothetical protein	-1.39
SO_2703	hypothetical protein	-1.66
**LambdaSo**		
SO_2939	hypothetical protein	1.10
SO_2960	hypothetical protein	1.43
SO_2963	prophage LambdaSo, HK97 family major capsid protein	1.19
SO_2964	ClpP protease family protein	1.29
SO_2965	prophage LambdaSo, HK97 family portal protein	1.34
SO_2969	prophage LambdaSo, holin, putative	2.21
SO_2970	hypothetical protein	1.81
SO_2971	hypothetical protein	2.02
SO_2972	hypothetical protein	1.91
SO_2974	hypothetical protein	2.76
SO_2976	hypothetical protein	-1.75
SO_3012	hypothetical protein	1.09
