# Supplementary material for: Transcriptome Analysis of Early Surface-Associated Growth of Shewanella oneidensis MR-1
Source: PLoS One. 2012 Jul 31;7(7):e42160. doi: 10.1371/journal.pone.0042160 (PMC3409153; doi:10.1371/journal.pone.0042160)
Supplement: Table S4 — Differentially regulated genes related to “metabolic adaptation” - Carbohydrate transport and metabolism. (PDF) [file pone.0042160.s006.pdf]

Table S4: Differentially regulated genes related to “metabolic adaptation” - Carbohydrate transport and metabolism

Locus	Gene	Product	log_2 ratio
SO_0345	*prpB*	2-methylisocitrate lyase	-1.58
SO_0525	-	drug resistance transporter, EmrB/QacA family protein	-1.54
SO_0538	*gapA-1*	glyceraldehyde-3-phosphate dehydrogenase	-1.43
SO_1505	-	transporter, putative	1.46
SO_1917	-	multidrug resistance protein, putative	-1.44
SO_2156	-	alpha amylase family protein	-1.27
SO_2347	*gapA-3*	glyceraldehyde-3-phosphate dehydrogenase	-2.88
SO_2389	*emrD*	multidrug resistance protein D	-1.77
SO_2486	*eda*	keto-hydroxyglutarate-aldolase/keto-deoxy-phosphogluconate aldolase	2.23
SO_2488	*pgl*	6-phosphogluconolactonase	2.34
SO_2489	*zwf*	glucose-6-phosphate 1-dehydrogenase	2.01
SO_2491	*pykA*	pyruvate kinase II	2.23
SO_2644	*ppsA*	phosphoenolpyruvate synthase	-1.15
SO_2970	-	hypothetical protein	1.81
SO_3547	*pgi*	glucose-6-phosphate isomerase	1.67
SO_3714	-	sugar-binding protein, putative	-1.05
SO_4040	-	integral membrane domain-containing protein	-1.55
