# Supplementary material for: Transcriptome Analysis of Early Surface-Associated Growth of Shewanella oneidensis MR-1
Source: PLoS One. 2012 Jul 31;7(7):e42160. doi: 10.1371/journal.pone.0042160 (PMC3409153; doi:10.1371/journal.pone.0042160)
Supplement: Table S5 — Differentially regulated genes related to “metabolic adaptation” - Lipid transport and metabolism. (PDF) [file pone.0042160.s007.pdf]

Table S5: Differentially regulated genes related to "metabolic adaptation" - Lipid transport and metabolism

Locus	Gene	Product	log_2 ratio
SO_0020	*fadA*	3-ketoacyl-CoA thiolase	-1.83
SO_0021	*fadB*	multifunctional fatty acid oxidation complex subunit alpha	-1.73
SO_0355	-	AMP-binding protein	1.64
SO_0496	-	hypothetical protein	1.31
SO_0572	-	enoyl-CoA hydratase	-1.02
SO_1677	*atoB*	acetyl-CoA acetyltransferase	-2.05
SO_1679	-	acyl-CoA dehydrogenase family protein	-2.31
SO_1680	-	enoyl-CoA hydratase	-2.30
SO_1681	-	enoyl-CoA hydratase/isomerase family protein	-1.78
SO_1891	-	3-oxoadipate CoA-succinyl transferase, beta subunit	-1.12
SO_1892	*atoD*	acetate CoA-transferase, subunit A	-1.78
SO_1894	-	acetyl-CoA carboxylase, biotin carboxylase, putative	-1.80
SO_1895	-	enoyl-CoA hydratase/isomerase family protein	-1.14
SO_1896	*pccB-1*	3-methylcrotonyl CoA carboxylase, beta subunit	-1.68
SO_1897	*ivd*	isovaleryl-CoA dehydrogenase	-2.16
SO_2395	-	acyl-CoA dehydrogenase family protein	-1.73
SO_2492	-	acyl-CoA dehydrogenase	-1.18
SO_2536	*fadE*	acyl-CoA dehydrogenase	-3.42
SO_2743	*acs*	acetyl-CoA synthetase	-2.22
SO_2768	-	acyl-CoA dehydrogenase family protein	-1.00
SO_2772	-	cytosolic long-chain acyl-CoA thioester hydrolase family protein	1.02
SO_3088	*fadJ*	multifunctional fatty acid oxidation complex subunit alpha	-1.41
SO_3089	*fadI*	3-ketoacyl-CoA thiolase	-2.25
SO_3263	-	3-oxoacyl-(acyl-carrier-protein) reductase, putative	1.08
SO_3265	-	hypothetical protein	1.61
