# Supplementary material for: Transcriptome Analysis of Early Surface-Associated Growth of Shewanella oneidensis MR-1
Source: PLoS One. 2012 Jul 31;7(7):e42160. doi: 10.1371/journal.pone.0042160 (PMC3409153; doi:10.1371/journal.pone.0042160)
Supplement: Table S6 — Differentially regulated genes related to “metabolic adaptation” - Nucleotide transport and metabolism. (PDF) [file pone.0042160.s008.pdf]

Table S6: Differentially regulated genes related to “metabolic adaptation” - Nucleotide transport and metabolism

Locus	Gene	Product	log_2 ratio
SO_0441	*purD*	phosphoribosylamine--glycine ligase	-1.51
SO_0442	*purH*	bifunctional phosphoribosylaminoimidazolecarboxamide formyltransferase	-1.80
SO_1301	*pyrB*	aspartate carbamoyltransferase	-1.38
SO_1513	-	hypothetical protein	-1.33
SO_2001	*ushA*	bifunctional UDP-sugar hydrolase/5'-nucleotidase periplasmic precursor	1.77
SO_2761	*purN*	phosphoribosylglycinamide formyltransferase	-1.07
SO_3554	*purE*	phosphoribosylaminoimidazole carboxylase, catalytic subunit	-1.42
SO_3555	*purK*	phosphoribosylaminoimidazole carboxylase ATPase subunit	-1.08
SO_3613	*purT*	phosphoribosylglycinamide formyltransferase 2	-3.15
SO_3705	-	5-methylthioadenosine nucleosidase/S-adenosylhomocysteine nucleosidase, put	-3.10
SO_3706	-	NupC family protein	-2.69
