# Supplementary material for: Transcriptome Analysis of Early Surface-Associated Growth of Shewanella oneidensis MR-1
Source: PLoS One. 2012 Jul 31;7(7):e42160. doi: 10.1371/journal.pone.0042160 (PMC3409153; doi:10.1371/journal.pone.0042160)
Supplement: Table S7 — Differentially regulated genes related to “metabolic adaptation” - Energy metabolism and related. (PDF) [file pone.0042160.s009.pdf]

Table S7: Differentially regulated genes related to "metabolic adaptation" - Energy metabolism and related

Locus	Gene	Product	log_2 ratio
**Energy production and conversion**			
SO_0101	*fdnG*	selenium-containing formate dehydrogenase, nitrate inducible, alpha subunit	-2.03
SO_0102	*fdnH*	formate dehydrogenase, nitrate-inducible, iron-sulfur subunit	-1.89
SO_0103	*fdnI*	formate dehydrogenase, nitrate-inducible, cytochrome b556 subunit	-2.13
SO_0107	*fdhD*	formate dehydrogenase accessory protein FdhD	-2.90
SO_0336	-	Na(+)/H(+) antiporter	-1.62
SO_0343	*acnA*	aconitate hydratase	-1.00
SO_0344	*prpC*	methylcitrate synthase	-1.61
SO_0396	*frdC*	fumarate reductase cytochrome b-556 subunit	-2.31
SO_0640	-	alcohol dehydrogenase, zinc-containing	1.24
SO_0714	-	monoheme cytochrome c	2.39
SO_0845	*napB*	cytochrome c-type protein NapB	-2.57
SO_0846	*napH*	quinol dehydrogenase membrane component	-2.25
SO_0847	*napG*	quinol dehydrogenase periplasmic component	-2.40
SO_0848	*napA*	nitrate reductase	-2.43
SO_0900	-	aldo/keto reductase family oxidoreductase	1.28
SO_0970	-	fumarate reductase flavoprotein subunit precursor	-1.00
SO_0988	-	formate dehydrogenase, alpha subunit	-1.86
SO_1014	*nuoI*	NADH dehydrogenase subunit I	1.11
SO_1020	*nuoB*	NADH dehydrogenase subunit B	1.13
SO_1232	*torA*	trimethylamine-N-oxide reductase	-1.00
SO_1251	-	ferredoxin, 4Fe-4S	-1.03
SO_1363	*hcp*	hydroxylamine reductase	-1.16
SO_1414	-	flavocytochrome c flavin subunit, putative	-2.54
SO_1427	-	decaheme cytochrome c	-3.22
SO_1663	*napF*	ferredoxin-type protein NapF	-1.14
SO_1678	*mmsA*	methylmalonate-semialdehyde dehydrogenase	-1.94
SO_1694	-	FAD-binding protein	-3.09
SO_1776	*mtrB*	outer membrane protein precursor MtrB	-1.24
SO_1778	*omcB*	decaheme cytochrome c	-1.08
SO_2096	-	hydrogenase expression/formation protein	-1.71
SO_2097	*hydC*	quinone-reactive Ni/Fe hydrogenase, cytochrome b subunit	-1.22
SO_2098	*hyaB*	quinone-reactive Ni/Fe hydrogenase, large subunit	-1.87
SO_2144	-	hypothetical protein	-1.33
SO_3369	-	hypothetical protein	1.09
SO_3371	-	cytochrome B561	1.36
SO_3392	-	oxidoreductase, FMN-binding	1.05
SO_3922	-	formate dehydrogenase, putative	-1.71
SO_4404	-	iron-sulfur cluster-binding protein	-1.36
SO_4469	-	alcohol dehydrogenase, iron-containing	1.19
SO_4480	*aldA*	aldehyde dehydrogenase	-1.96
SO_4483	-	cytochrome b, putative	-1.62
SO_4513	-	formate dehydrogenase, alpha subunit	-1.59
SO_4514	*fdhB-2*	formate dehydrogenase, iron-sulfur subunit	-1.44
SO_4515	-	formate dehydrogenase, C subunit, putative	-1.16
SO_4606	-	cytochrome c oxidase, subunit II	-3.17
