# Supplementary material for: Transcriptome Analysis of Early Surface-Associated Growth of Shewanella oneidensis MR-1
Source: PLoS One. 2012 Jul 31;7(7):e42160. doi: 10.1371/journal.pone.0042160 (PMC3409153; doi:10.1371/journal.pone.0042160)
Supplement: Table S8 — Sequence of primers used in this study. (DOCX) [file pone.0042160.s010.docx]

**Table S8:** Sequence of primers used in this study

| **Primer name** | **Sequence (5’ to 3’)** |  |
| --- | --- | --- |
| SO2500-qPCR-fw | CGGCAATCGAACACAACA |  |
| SO2500-qPCR-rev | **CGCATCGAGTTCTTTTTGC** |  |
|  |  |  |
| SO3376-qPCR-fw | GATGCTTAGCCTATTATCGAGTTGTT |  |
| SO3376-qPCR-rev | **GCCTTAAGCGGCCCATTA** |  |
|  |  |  |
| SO2660-qPCR-fw | cggtcacgcagtaccaga |  |
| SO2660-qPCR -rev | **gtggccttgcatttctttg** |  |
|  |  |  |
| recA-qPCR-fw | **TCACATCAACCGCACCAGAACG** |  |
| recA-qPCR-rev | **CGCTCTTGATCCTATCTACGCG** |  |
|  | | |
